# Characterizing co-purchased food products with soda, fresh fruits, and fresh vegetables using loyalty card purchasing data in Montréal, Canada, 2015–2017

**DOI:** 10.1186/s12966-024-01701-8

**Published:** 2025-02-17

**Authors:** Hiroshi Mamiya, Kody Crowell, Catherine L. Mah, Amélie Quesnel-Vallée, Aman Verma, David L. Buckeridge

**Affiliations:** 1 Department of Epidemiology, Biostatistics, and Occupational Health, Faculty of Medicine, McGill University, Suite 1200, 2001 McGill College Avenue, Montréal, Québec H3A 1G1 Canada; 2 School of Health Administration, Faculty of Health, Dalhousie University, Halifax, Canada; 3 Department of Sociology, McGill University, Montréal, Canada

**Keywords:** Association rule mining, Supermarket loyalty card data, Nutrition surveillance, Dietary pattern analysis, Public health nutrition

## Abstract

**Background:**

Foods are not purchased in isolation but are normally co-purchased with other food products. The patterns of co-purchasing associations across a large number of food products have been rarely explored to date. Knowledge of such co-purchasing patterns will help evaluate nutrition interventions that might affect the purchasing of multiple food items while providing insights about food marketing activities that target multiple food items simultaneously.

**Objective:**

To quantify the association of food products purchased with each of three food categories of public health importance: soda, fresh fruits and fresh vegetables using Association Rule Mining (ARM) followed by longitudinal regression analysis.

**Methods:**

We obtained transaction data containing grocery purchasing baskets (lists of purchased products) collected from loyalty club members in a major supermarket chain between 2015 and 2017 in Montréal, Canada. There were 72 food groups in these data. ARM was applied to identify food categories co-purchased with soda, fresh fruits, and fresh vegetables. A subset of co-purchasing associations identified by ARM was further tested by confirmatory logistic regression models controlling for potential confounders of the associations and correlated purchasing patterns within shoppers.

**Results:**

We analyzed 1,692,716 baskets. Salty snacks showed the strongest co-purchasing association with soda (Relative Risk [RR] = 2.07, 95% Confidence Interval [CI]: 2.06, 2.09). Sweet snacks/candies (RR = 1.73, 95%CI: 1.72–1.74) and juices/drinks (RR:1.71, 95%CI:1.71–1.73) also showed strong co-purchasing associations with soda. Fresh vegetables and fruits showed considerably different patterns of co-purchasing associations from those of soda, with pre-made salad and stir fry showing a strong association (RR = 3.78, 95% CI:3.74–3.82 for fresh vegetables and RR = 2.79, 95%CI:2.76–2.81 for fresh fruits). The longitudinal regression analysis confirmed these associations after adjustment for the confounders, although the associations were weaker in magnitude.

**Conclusions:**

Quantifying the interdependence of food products within shopping baskets provides novel insights for developing nutrition surveillance and interventions targeting multiple food categories while motivating research to identify drivers of such co-purchasing. ARM is a useful analytical approach to identify such cross-food associations from retail transaction data when combined with confirmatory regression analysis to adjust for confounders of such associations.

**Supplementary Information:**

The online version contains supplementary material available at 10.1186/s12966-024-01701-8.

## Background

Unhealthy diets are major preventable risk factors for chronic diseases, including type II diabetes mellitus, some types of cancers, and cardiovascular diseases [1–4]. National health surveys routinely collect the dietary records of key food categories that influence the healthiness of diets, most commonly sugar-sweetened beverages, fresh fruits, and fresh vegetables [5, 6]. These food categories are also frequently discussed as the target of nutrition interventions, including taxation and subsidization [7, 8].

Food products are purchased in combination rather than in isolation [9, 10]. Co-purchasing is a frequent appearance of certain food products together in shopping baskets beyond chance, often because the products constitute the same meal recipe consumed simultaneously (e.g., soda and chips, chips and dips, and eggs, milk and pancake mix) or marketed together, for example, displayed adjacent to each other in stores [11, 12]. Identifying co-purchasing patterns provides insights about previously unknown drivers of these purchases, including simultaneous marketing of multiple food items. Also, knowledge about cross-product associations allows the monitoring of the “spillover effect” of an intervention, i.e., changes in the sales of food groups in response to an intervention promoting or discouraging the sales of another food group, if the two groups are dependent, or co-purchased [13].

Studies investigating co-purchasing patterns of food products in population health research are scarce, and such patterns are likely to vary across populations [14–16]. Grocery transaction data provide a list of purchased food times at each transaction (i.e., shopping basket) and thus allow learning such associations when combined with analytical methods that identify associations among hundreds of variables (food products). Association Rule Mining (ARM), also known as market basket analysis, is one such method that rapidly estimates associations between variables using computationally efficient algorithms [17–19]. Biomedical and population health researchers have begun utilizing ARM to identify associations among a rapidly growing number of variables representing diagnosis, comorbidities, treatments, and behavioral and environmental risk factors [17, 20–24].

ARM is distinct from and complementary to widely used dietary or purchasing pattern analyses, a collection of methods to summarize the consumption or purchasing of multiple food products into data-driven typologies (e.g., *Western* or *Mediterranean* dietary pattern). These patterns are learned from statistical dimensionality-reduction techniques, such as factor analysis, Principal Component Analysis and clustering [15, 25–27]. Dietary pattern analyses allow for predicting chronic disease outcomes and classifying people based on the overall similarity of food products they consume or purchase. However, they do not explicitly quantify the strength of co-purchasing associations across these items. ARM estimates the strength of such co-purchasing associations within shopping baskets (or co-consumption within a meal occasion).

The primary objective of this study is to identify food categories that are co-purchased with three food categories of public health importance: soda, fresh fruits, and fresh vegetables (not including canned or frozen produce). To estimate these co-purchasing associations, we applied ARM to grocery transaction data in a loyalty program database in one of the major supermarket chains in Montréal, Canada. Soda in this study is defined as carbonated soft drinks containing sugar and those containing artificial sweeteners (diet soda). Vegetables and fruits are the key elements of dietary guidelines associated with a reduced risk of cardiovascular diseases cancers and all-cause mortality [2, 3, 28, 29]. Associations estimated by ARM are not confounder-controlled. Therefore, as a secondary objective, we applied a confirmatory longitudinal regression model controlling for potential time-fixed and time-varying confounders of the associations and the correlation of purchasing within cardholders to a subset of associations identified by ARM.

## Methods

This is an observational study analyzing shopping baskets, which are the time-stamped lists of purchased food products between February 1, 2015, and September 30, 2017. These data represent longitudinal shopping records among the members of the loyalty card program (hereafter called *cardholders*) in a supermarket retail chain. The cardholders were the residents of the Island of Montréal, Quebec, Canada. Montréal is a metropolitan area encompassing a population of 1.7 million residents in 2016 [30]. Additionally, the same retailer provided non-longitudinal basket data representing transactions from non-cardholders. While these non-longitudinal (non-cardholder) data do not allow confirmatory longitudinal regression analysis, we applied ARM to them as a sensitivity analysis, as detailed in Methods. We note that ARM identifies purchasing associations at the transaction level: the unit of analysis in this study is a shopping basket rather than a person. This is in contrast to dietary pattern analyses that summarize the pattern of diets within each person (as opposed to basket) [31]. The retail chain providing these data is not a discount chain selling food with competitive pricing, nor an up-scale chain targeting customers in a high-income segment.

The retailer is one of seven major supermarket chains operating in Quebec during the study period. In terms of market share, its proportion of dollar sales for soda ranged between 5 and 10% of the sales of soda among the six remaining competing supermarket chains, two supercenter chains, four pharmacy chains, and three convenience store chains in Montréal in 2013, as calculated by store-level sales data from a previous study [32]. As for fresh fruits and fresh vegetables, our retailer’s market share was 10–20% among the supermarket and supercenter chains selling produce (convenience stores and pharmacies were excluded from the denominator, as they do not typically sell much produce).

There were 20–50 stores belonging to this chain in Montréal. We provide ranges rather than the precise number of stores and the proportions of sales to maintain the anonymity of the retailer. Geographic coverage of stores belonging to this chain is as follows: 2,723 out of 3,026, and all 3,026 census Dissemination Area (DA) in Montréal physically overlapped with a circular buffer centered around these stores, with a 3 km and 5 km radius, respectively. DAs represent the smallest census geographic unit in Canada for which census data are disseminated and contain 400–700 residents.

### Basket and cardholder data

Individual baskets contain a list of purchased products and the corresponding product-specific Universal Product Code, and quantity purchased, as barcode-scanned at the time of purchasing. We linked the basket data to cardholders through hash-anonymized card IDs. The only information available from the cardholder database, aside from basket data, is self-reported Canadian postal codes as their residential location, which were converted to DA through the Canadian Postal Codes Conversion Plus File and linked to aggregated (ecological) DA-level socio-economic and demographic attributes measured by the 2016 Canadian Census [30]. While not possible to verify, a single loyalty card is likely to be shared by members of the same household. We also note that this is an open cohort without a well-defined follow-up time (e.g., cardholders can re-visit the store after prolonged months of non-transaction).

### Exclusion of cardholders

From 1,343,470 cardholders in the province of Quebec, we selected those residing in Montréal. We then removed cardholders in DAs whose census information was suppressed due to confidentiality. We also excluded cardholders who may have been transient residents of Montréal, as evaluated by infrequent shopping trips (equal to or less than six baskets per year). From the remaining 251,246 cardholders, we randomly sampled 15,000 cardholders that contained 1,728,476 baskets as the study sample. The sample was split into 12,000 (1,355,875) cardholders for ARM (primary objective) and the remaining 3,000 (372,601 baskets) cardholders for the confirmatory regression (secondary objective). Since multiple statistical tests on the same data may increase the risk of false positives, we split the sample to avoid using the same data for hypothesis generation with ARM and subsequent confirmatory analysis. We reduced the sample size to 15,000 cardholders from the original 251,246 cardholders to reduce computational overhead, as increasing the size further may provide little benefit in terms of precision. In fact, the width of the regression-estimated Confidence Intervals (CIs) (i.e., precision) based on the 3000 participants in the confirmatory analysis is extremely small, as seen in our results below, while model fitting and the estimation of profile CI from the fitted models took approximately 13 min per model.

### Exclusion of baskets

**Fig. 1:**
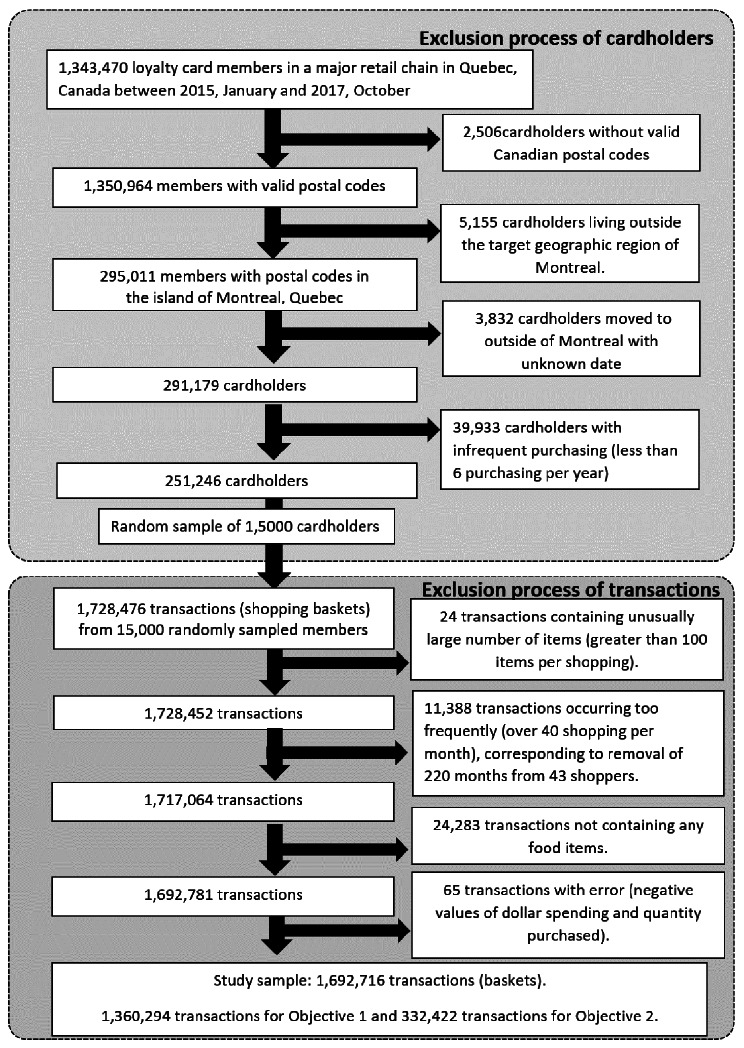
Flowchart describing exclusion of cardholders and transactions

From the 1,728,476 baskets, we excluded extremely large baskets (containing more than 100 products) and baskets whose monthly occurrence was unusually high (over 40 baskets per month per cardholder), which together led to the exclusion of a small fraction (2%) of all baskets. We further removed baskets that did not contain any food products. We excluded negative values of dollar spending, which indicate a refund due to product return or the return of recyclable containers and bottles. The exclusion process and the final analytical sample are summarized in Fig. 1.

**Fig. 2:**
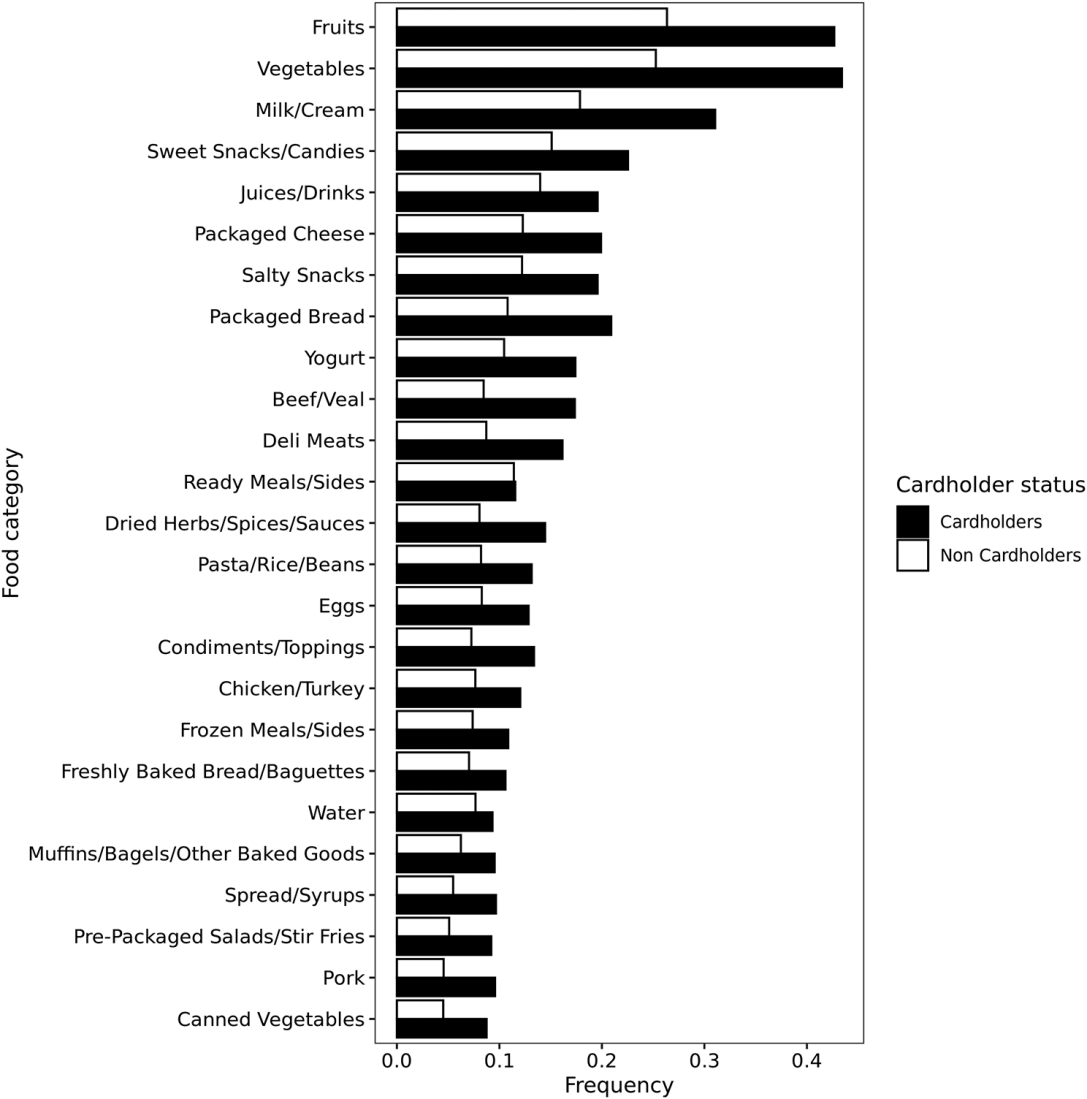
Frequency of top 25 food categories in shopper baskets. Frequency indicates the incidental purchasing of categories in each basket rather than the number of units purchased, thus counted as one even if multiple units of the same category were purchased. The top two categories indicate fresh fruits and fresh vegetables, not canned or frozen products

The median number of products per basket was 12 (IQR:6–24), 19 (IQR:8–52), and 16 (IQR:7–40) for baskets containing soda, fresh vegetables, and fresh fruits, respectively (Additional file 1, Supplementary Table S1), indicating that shopping baskets containing soda had a slightly smaller number of items in baskets.

#### Description of cardholders

The area-level socio-economic characteristics of the sampled 15,000 cardholders were nearly identical to those of all 251,246 cardholders in Montréal (Additional file 1, Supplementary Table S2). Relative to the general population in Montréal, cardholders had a higher area-level percent of residents not completing a high-school diploma, (Median = 16.2%, IQR:9.8–24.0% vs. Median = 13.5%, IQR: 8.1–20.7% for the general population and cardholders, respectively) and immigrants (Median = 31.4%, IQR: 20.6–44.9 vs. Median = 26.8%, IQR: 18.3–38.7%).

#### Co-purchasing associations

Table 3 lists the food categories that showed strong association and high support with soda, fresh vegetables, and fresh fruits. The value of support indicates the frequency (in percent) of the two categories appearing together among all baskets. Salty snacks showed the strongest association with soda and high support (i.e., the co-purchasing frequency with soda). Water also showed a strong association but low support, relative to that of sweet snacks/candies, and juices/drinks. Supplementary Figs. S1–S3 are forest plots containing larger lists showing the top 25 co-purchased categories with soda, fresh vegetables, and fresh fruits, respectively. The top 25 co-purchased food categories with soda differed noticeably from those associated with fresh vegetables and fresh fruits. For example, fresh fish and frozen fish/seafood were among the co-purchased categories with fresh vegetables and fruits but were absent from the soda-associated list. Conversely, frozen meals/sides and ready meals/sides ranked within the top 25 co-purchased categories with soda but did not appear among the top 25 categories associated with fresh vegetables and fruits.

### Food products and categories

Approximately 40,000 unique food products, as defined by their product UPC, were grouped into 72 food categories defined by the retailer listed in Table 1. Thus, not all categories aligned with the nutritionally relevant classifications or profiling of food groups based on nutritional compositions [33–35]. This is because nutritionally relevant classification or profiling required matching product UPCs with external product databases that were unavailable at the time of the study. In addition, basket analysis using ARM defines a product based on purchasing unit rather than standardized volume or weight, implying that a product consisting of a bottle of soda and a product packed with 12 bottles were equally considered as a single item of soda.

**Table 1:** List of food categories, with the corresponding number of individual food products in range. Exploratory analysis of grocery purchasing patterns using loyalty card grocery purchasing data from a grocery retail chain in Montréal, Canada, 2015–2017

Department	Category	Number of products^a^	Category description
Beverages	Beer/Cider	1000–2000	
	Coffee	500–1000	Coffee beans, ground or whole
	Drink Mixes	100–500	Flavored liquid water enhancer, powered fruits and milk drinks, liquid syrup, non-alcohol cocktails, all containing artificially added sugar
	Iced Tea Coffee	50–100	Coffee or tea mixes with artificially added sugars or artificial sweeteners
	Juices/Drinks	500–1000	100% fruits juice and drinks (not 100%), smoothies, nectars, and sparkling juices. Refrigerated and non-refrigerated.
	Soda	100–500	Carbonated soft drinks containing sugar or artificial sweeteners (e.g., diet products).
	Soy/Rice/Nut Beverages	100–500	Products with and without artificially added sugar and sodium.
	Sports Energy Drinks	100–500	
	Tea/Hot Drinks	100,500	Dried tea leaves and a few powered hot chocolates with artificially added sugar
	Water	100,500	Non-sweetened sparkling and tonic water, bottled water, flavored water, and nutrient-enhanced flavored water sweetened with sugar.
	Wines/Cocktails/Coolers	500,1000	
Bread-Bakery-Products	Buns/Rolls	100,500	
	Chilled Desserts/Dough	100,500	
	Desserts/Pastries	500,1000	
	Freshly Baked Bread/Baguettes	100,500	
	Muffins/Bagels/Other Baked Goods	100,500	
	Packaged Bread	100,500	
	Tortillas/Flat Breads	100,500	
Cereals	Cereal Bar	100,500	
	Cereals	100,500	Mix of sugar-sweetened and non-sweetened cereals
Dairy-Cheese	Butter/Margarine	50,100	
	Deli Cheese	100,500	Mostly block, wedge, and round-shaped cheese, including locally produced cheese. Unlike processed cheese that tended to be placed adjacent to butter/margarine, deli cheese was located near the deli counter.
	Eggs	50,100	
	Milk/Cream	100,500	
	Packaged Cheese	1000,2000	Thinly sliced cheese wrapped by plastic films, shredded cheese, bottled solid or semi-solid cheese, cheese spread, and cream cheese.
	Sour Cream	100,500	
	Yogurt	500,1000	Mix of plain (unsweetened) and flavored (sugar-sweetened) solid and liquid yogurt
Deli-Prepared-Meals	Antipasto/Dips/Pates	100,500	Dips, spreads, pate/cretons, and olives
	Deli Meats	500,1000	Mix of processed and unprocessed meats.
	Ready Meals/Sides	500,1000	Deli-prepared salads, sushi, desserts, pasta, meat and fish means, soups, and appetizers
Fish-Seafood	Fresh Fish	100,500	
	Fresh Seafood	100,500	
Frozen-Food	Frozen Appetizers Snacks	50,100	
	Frozen Bakery	100,500	
	Frozen Beverages	50,100	
	Frozen Fish/Seafood	100,500	
	Frozen Fruits	50,100	
	Frozen Meals/Sides	500,1000	
	Frozen Meat/Poultry	100,500	
	Frozen Vegetables	100,500	
	Ice Cream/Frozen Confections	500,1000	
Fruits	Dried Fruits	50,100	
	Fruits	500,1000	Fresh fruits
Grocery	Baking Ingredients	500,1000	
	Canned Fruits	50,100	
	Canned Meal	1,50	
	Canned Meat	1,50	
	Canned Other Food	1,50	
	Canned Seafood	100,500	
	Canned Soup	100,500	
	Canned Vegetables	100,500	
	Spreads/Syrups	100,500	Peanuts butters, jams, and other sweetened spreads.
	Condiments/Toppings	1000,2000	
	Dried Herbs/Spices/Sauces	1000,2000	
	Ethnic Food	100,500	Dried or bottled food in Asian, Indian, Latin, and Mediterranean style.
	Oils/Vinegars	100,500	
	Pasta/Rice/Beans	1000,2000	Mix of refined and unrefined products.
Meat-Poultry	Beef/Veal	100,500	Unprocessed meats.
	Chicken/Turkey	100,500	Unprocessed meats.
	Lamb Horse Game Meat	50,100	Unprocessed meats.
	Pork	100,500	Unprocessed meats.
	Rabbit Fowl	1,50	Unprocessed meats.
	Sausages Bacon Gluten Free	1,50	
	Sausages/Bacon	100,500	
Snacks	Nuts/Seeds/Dried fruit	500,1000	
	Salty Snacks	1000,2000	Mostly potato chips, but also include popcorns, crackers, salted nuts, rice snacks, and jerky.
	Sweet Snacks/Candies	3000,5000	Candies, gums, chocolate, pudding, cookies and cakes, and chewy bards
Spreads-Syrups	Spread/Syrups	100,500	
	Vegan/Vegetarian Food	100,500	Variations of tofu product and a small number of legume-based meat substitute
Vegetables	Fresh Herbs	100,500	
	Vegetables	1000,2000	Fresh vegetables
	Pre-Packaged Salads/Stir Fries	100,500	Uncooked and pre-cut vegetables to be consumed as a salad or heated as stir-fry vegetables

Abbreviation: UPC, Universal Product Code

^a^ Precise number of individual products within categories is not shown to maintain the anonymity of the retailer

### Statistical analysis

#### Association rule mining

While traditional dietary pattern analyses group food items into clusters but do not explicitly quantify the pairwise associations between food groups, ARM quantifies such associations. Specifically, ARM estimates the strength of the relationship “customers who choose product X also choose a product Y”. The variables X and Y are commonly termed *antecedent* and *consequent*, respectively, and the antecedent in this study is soda, fresh vegetables, or fresh fruits. There is no time-ordering for X and Y; the association represents cross-sectional (undirected) associations within baskets. ARM generates a metric called lift to describe the associations [36]. Let soda be the antecedent and chocolate be the consequent and let the frequency (prevalence) of baskets containing soda be denoted by P(soda) and the frequency of chocolate be denoted by P(chocolate). Then, the conditional probability of selecting chocolate given soda is P(chocolate | soda), and the joint probability, i.e., the prevalence of baskets containing the antecedent and consequent, is P(soda, chocolate). Lift is then computed as:

Lift(soda, chocolate) = P(soda, chocolate)/(P[soda]*P[chocolate]).

Thus, the lift is interpreted as the strength of association beyond chance alone, i.e., adjusted for the baseline probabilities P(soda) and P(chocolate). Values of lift greater than 1.0 indicate that the products are co-purchased beyond chance alone, and the null value of 1.0 indicates the lack of associations.

We implemented ARM using the a priori search algorithm in the *arules* package in R statistical software [37]. We estimated the lift of associations between the 72 food categories (consequent) and each of our target categories of interest: soft drinks, fresh vegetables, and fresh fruits (antecedent) from a single ARM model fit to the 12,000 cardholders (thus, we did not fit the model 3 times to the same data). While lift is a commonly used measure of association in data mining, it is an unfamiliar concept in health science. We thus converted the estimated values of lift into an epidemiologically relevant measure of the strength of associations, the Risk Ratio (RR). The interpretation of RR is as follows: The probability of purchasing the consequent item (chocolate) given the antecedent (e.g., soda) divided by the probability of purchasing chocolate when the antecedent is not purchased, i.e., P(chocolate | soda)/P(chocolate | no soda). As in lift, RR (chocolate, soda) = 1 indicates the lack of co-purchasing associations (null associations). The values of RR are more extreme than those of lift (i.e., further away from 1), and the deviation increases as the values of P(chocolate) and lift increase.

A detailed description of lift and its relationship with RR is provided by a recent review [36]. To reduce computational burden and generate an excessively large number of weak associations, ARM requires two user-defined inputs to rule out highly infrequent and weak association pairs, which are minimum support and minimum confidence. We set minimum support to 0.01 (1%) and minimum confidence to 0.05 (5%). The description of these inputs is provided in Appendix S1 and by a previous review of ARM [36].

#### Confirmatory longitudinal analysis to obtain adjusted co-purchasing associations

As ARM is a data-mining algorithm for hypothesis generation, it estimates *crude* associations not adjusted for potential confounders between two food categories. Additionally, ARM treats baskets as independent observations not repeated within cardholders, thus underestimating standard errors, i.e., falsely narrower CI. Therefore, after running ARM, we re-estimated associations for three consequent food categories having strong associations with the target categories (antecedents: soda, fresh vegetables, and fresh fruits) using logistic regression models with random intercepts, including potential confounders. We thus fitted nine models in total. Specifically, we used logistic regression with cardholder-specific random intercepts with an autoregressive order 1 correlation structure. The models also contained the binary purchasing status of each of the antecedent food categories (exposure) and the binary status of each of the consequent categories (outcome), in addition to the confounders described below. The odds ratio is the default interpretation of (exponentiated) regression coefficients in logistic regression models. However, to enhance the ease of interpretation, we converted odds radio into RR using the regression standardizing method [38] combined with the delta method [39]. We fitted these logistic regression models with random intercepts using the nlme package in R statistical software [40].

#### Confounders

Potential confounders for the regression analysis included basket size as the total number of products [12, 41]. We also added indicator variables for 7 days preceding national and provincial holidays to account for potentially differing purchasing during pre-holiday periods. Since cardholders’ socio-economic and demographic characteristics were unavailable, we used area-level characteristics at the level of DA measured by the 2016 Canadian census. These ecological variables are the proportion of residents not completing a high-school diploma, employment among the labor force, immigrants, the mean age of residents, mean family size, and median family income. The selection of confounders was determined by model fit measured by Akaike’s Information Criterion.

#### Sensitivity analyses

As a sensitivity analysis of the regression modelling, we investigated the effect measure modification (heterogeneity) of the associations across the DA-level education and income, specifically by adding an interaction term between exposure (purchasing of soda, fresh fruits, or fresh vegetables) and each of area-level income and education. The income and education variables were standardized (mean centered and scaled by one standard deviation) to improve the convergence of the random intercept logistic regression models. Thus, the interaction terms represent the change in odds associated with the exposure (binary purchasing) variables and a one standard deviation increase in terms of area-level income or education. We report the odds ratio estimates of these interaction terms, as the computation of standard errors for the coefficient of interaction terms from logistic regression was straightforward. Because basket compositions could differ between cardholders and non-cardholders [42], we also applied ARM to non-cardholders’ baskets (non-cardholders generated 12.3 million baskets, relative to 7.4 million generated by all cardholders in Montréal). However, longitudinal regression analyses were not applied to these data without longitudinal linkage of baskets. Finally, we also applied ARM to a subset of cardholders consisting of frequent users of the target supermarket chain (as opposed to infrequent or non-loyal shoppers using other supermarket chains). Frequent users were determined to be those spending at least 514 Canadian dollars monthly, based on the annual median food expenditure of one-person households estimated from the Survey of Household Expenditure, 2019 [43]. Codes to perform the analyses and prepare data are available publicly https://github.com/hiroshimamiya/grocery_arm. Consent for the secondary use of loyalty card data for analyzing consumer behavior was initially obtained by the retailer when shoppers subscribed to the loyalty program. Directly obtaining informed consent for this specific secondary analysis was waived by the Institutional Review Board (IRB) at the Faculty of Medicine, McGill University (IRB approval # A01-E03-13B), as the study complied with Article 5.5 A and Article 5.5B of the Tri-Council Policy Statement: Ethical Conduct for Research Involving Humans, regarding Consent and Secondary Use of Information for Research Purposes, Privacy, and Confidentiality [44].

## Results

### Description of baskets

The sample of 15,000 cardholders contained 1,692,716 baskets after the exclusion criteria, with 1,360,294 baskets from 12,000 cardholders for ARM (Objective 1) and the remaining 332,422 baskets from 3,000 cardholders for Objective 2 (Fig. 1). The median number of purchased products per basket was 6 (Interquartile Rane [IQR]:3–12) (Table 2), and the median number of baskets, i.e., transaction per shopper-month, was 4 (IQR:2–7). Fresh fruits and fresh vegetables were the most commonly purchased categories in terms of proportion, appearing in over 40% of baskets, while the percentage of baskets containing soda was approximately 10% (Fig. 2).

**Table 2:** Summary of baskets between members and non-members

Basket summary	Cardholders		Non-cardholders		
	Median	IQR		Median	IQR
Number of categories ^a^	5.0	2.0–8.0		3.0	1.0–5.0
Product quantities ^b^	6.0	3.0–12.0		3.0	2.0–6.0
Dollar spending	25.2	12.5–49.5		14.2	6.7–28.1

Abbreviation: IQR, interquartile range

^a^ Number of distinct categories within baskets

^b^ Number of purchased food products within baskets

Basket represents a list of items purchased in a single transaction (shopping trip)

**Table 3:** Relative risk of purchasing the consequent food category given the purchasing of the antecedent food category estimated by Association Rule Mining and confirmatory logistic regression models with random intercepts

Antecedent	Consequent	Support	RR (95%CI) from ARM	RR (95%CI) from Regression
Soda	Salty snacks	3.20%	2.07 (2.06, 2.09)	1.54 (1.52, 1.57)
Soda	Sweet snacks/candies	3.17%	1.73 (1.72, 1.74)	1.20 (1.18, 1.22)
Soda	Juices/drinks	2.73%	1.71 (1.71, 1.73)	1.27 (1.25, 1.29)
Soda	Water	1.50%	1.98 (1.96, 2.01)	NA
Fresh vegetables	Pre-packaged salads/stir fries	6.85%	3.78 (3.74, 3.82)	2.20 (2.16, 2.24)
Fresh vegetables	Canned vegetables	6.08%	2.98 (2.94, 3.01)	1.63 (1.61, 1.66)
Fresh vegetables	Deli cheese	5.74%	3.00 (2.97, 3.04)	1.47 (1.44, 1.49)
Fresh vegetables	Fresh herbs	3.44%	6.56 (6.43, 6.70)	NA
Fresh fruits	Pre-packaged salads/stir fries	6.22%	2.79 (2.76, 2.81)	1.67 (1.64, 1.70)
Fresh fruits	Cereals	5.71%	2.56 (2.20, 2.58)	1.45 (1.42, 1.47)
Fresh fruits	Yogurt	11.47%	2.59 (2.58, 2.61)	1.54 (1.52, 1.56)
Fresh fruits	Nuts/seeds/dried fruits	3.64%	2.72 (2.69, 2.76)	NA

Abbreviations; Relative Risk: RR, ARM: Association Rule Mining

For the regression analysis, the outcome variable was the binary purchasing status of the consequent food category, and the exposure variable was the antecedent food category

NA indicates the co-purchasing association not investigated by the regression analysis due to a smaller value of relative risk or support compared to other food categories

For co-purchasing patterns of fresh vegetables, fresh herbs showed a strong association but low support relative to other categories (Table 3 and Supplementary Fig. S2). As well, pre-packaged salads/stir fries, canned vegetables, and deli cheese were also strongly associated with fresh vegetables. As for the co-purchasing patterns of fresh fruits (Table 3, Supplementary Fig. S3), fresh herbs again showed a strong association but with low support, followed by pre-packaged salads/stir fries, Yogurt and cereals. Nuts/seeds/dried fruits category also showed a strong association, albeit at a considerably lower frequency.

#### Longitudinal analysis

The confounder-adjusted RR estimated by the logistic regression models (Table 3) are statistically significant, as indicated by 95% CIs that do not include the null value of 1.00, equivalent to a p-value below 0.05. However, the regression-estimated RRs are consistently closer to the null value (RR = 1.0) compared to the unadjusted associations estimated by ARM. All the estimates showed narrow 95% CIs, reflecting the large sample size (332,422 transactions from 3,000 shoppers), which yielded highly precise estimates.

#### Sensitivity analyses

Many interactions between the antecedent categories and each of the DA-level education and income variables were statistically conclusive i.e., p-values are less than the critical value of 0.05, likely due to the large sample size (Supplementary Tables S3–S5). However, the magnitude of the joint effects was generally small. For example, an increase of one standard deviation in the area-level proportion of residents without high school diplomas was associated with only 1.04 higher odds of purchasing sweet snacks and candies when soda was purchased (seventh row in Supplementary Table S3). Thus, the effect measure modification (interaction) of these co-purchasing associations by area-level income and education is nearly negligible in magnitude.

ARM applied to non-cardholders’ baskets show similar patterns of co-purchasing to those of cardholders for soda (Supplementary Fig. S4). Corresponding analysis of fresh vegetables (Supplementary Fig. S5) and fresh fruits (Supplementary Fig. S6) also showed somewhat comparable patterns of co-purchasing between cardholders and non-cardholders. When ARM was applied to “more loyal” cardholders whose monthly spending was greater than 514 Canadian dollars, the ranking of co-purchased food products with soda changed slightly. However, salty snacks, sweetened snacks and candies, and juices and drinks still showed high co-purchasing frequency and associations with soda as in the main analysis (Supplementary Fig. S7). For fresh vegetables and fresh fruits, the ranking of co-purchased categories was largely similar to those of the main analysis (Supplementary Figs. S8 and S9).

## Discussion

We used loyalty card grocery transaction data to investigate food categories co-purchased with soda, fresh vegetables, and fresh fruits in Montréal, Canada. Our findings showed that soda purchases were commonly associated with salty snacks, sweet snacks/candies, and juices/drinks within the same shopping baskets. In contrast, fresh vegetables had strong co-purchasing associations with pre-packaged salads/stir-fries, canned vegetables, and deli cheese. Fresh fruits were also frequently co-purchased with pre-packaged salads/stir-fries in addition to cereals and yogurt. To confirm these associations, we used longitudinal regression models that accounted for within-shopper correlations of shopping baskets and potential confounders. While the regression models confirmed these co-purchasing associations, the RRs were somewhat lower than those obtained from ARM.

The food categories frequently co-purchased with soda align with findings from prior dietary pattern analyses. Most studies have identified ‘unhealthy’ latent dietary patterns, often named as *Western*, *sweets*,* snack and high-fat*, or *high-convenience* dietary patterns [25, 45–47]. While the specific food group composition varies slightly across studies and populations, these patterns are generally characterized by the inclusion of sugar-sweetened beverages including soda, high-sodium foods including salty snacks, sugar confectionaries, red meat, ready-made meals, fast foods, and processed food products based on refined grains rather than whole grains. Additionally, previous studies examining the association between beverage types and non-beverage dietary patterns suggest that soda is a strong predictor of unhealthy dietary patterns [10, 48]. Many food categories included in the previously reported unhealthy dietary patterns appear among the top 25 items co-purchased with soda in our study, with the exception of fast food items, which are not typically present in supermarkets. The strong co-purchasing association between soda and salty snacks may be partly due to the complementary nature of these two categories, since salty foods increase the consumption of fluids including soda, and soda has been shown to heighten cravings for salty foods [49, 50].

While we found a strong co-purchasing association between juices/drinks and soda, previous studies suggest that only sugar-sweetened fruit drinks, rather than 100% fruit juices, co-occur with soda within unhealthy dietary patterns [10, 48]. However, our retailer-defined categories do not differentiate fruit drinks from 100% juices. Similarly, our categorization does not separate flavored and often sugar-sweetened water from plain, unsweetened water. This limitation in categorization from a nutritional standpoint may have contributed to the strong co-purchasing association between soda and water in our study, which is inconsistent with prior findings: an inverse relationship between plain water and sugar-sweetened beverages, including soda [10, 51].

Our findings regarding co-purchased food groups with fresh vegetables and fruits are consistent with previous studies, which grouped fresh fruits, fresh vegetables, and their co-purchased food categories into ‘healthy’ dietary patterns. These patterns are often labeled as *prudent*, *fiber-rich*, *low-convenience*, or *low-fat* pattern and typically include nuts and seeds, whole grains, fish, poultry, legumes, plain water, unsweetened tea or coffee, low-fat milk, yogurt, fresh (deli, unprocessed) cheese, artificially sweetened i.e., diet or zero-sugar, beverages, and 100% fruit and vegetable juices, in addition to fresh vegetables and fresh fruits [45–47]. Many of the previously identified ‘healthy’ categories appeared among the top 25 co-purchased items with fresh fruits and fresh vegetables identified by ARM.Pre-packaged salads/stir-fries showed the strongest co-purchasing association with fresh vegetables in our study. However, these packaged products do not necessarily align with previously identified *salad and olive oil* or *salad vegetables* dietary patterns that consist of raw vegetables and the minimally processed ingredients such as olive oil [52, 53]. This is because the pre-packaged food category in our data contains products with leafy vegetables with dressings, which may be high in sodium.

The strong co-purchasing association between canned vegetables and fresh vegetables observed in our study aligns with findings from a previous study, which reported that frequent consumers of canned vegetables had a 20% higher frequency of consuming fresh vegetables compared to infrequent consumers [54]. Additionally, we found slight differences in the food categories co-purchased with fresh fruits compared to those with fresh vegetables. The food categories associated with fresh fruits align with previously reported fiber-rich *cold-food* and *prudent breakfast* patterns. These categories include nuts and seeds, milk, yogurt, fresh cheese, and unsweetened, unprocessed cereals [55, 56]. However, our study is unable to distinguish unsweetened cereals from sweetened cereals.

While our findings based on ARM generally align with those from dietary pattern analyses, there are notable differences in the interpretation of findings. First, our results reflect co-purchasing associations within grocery baskets, whereas dietary pattern analysis provides insights into the grouping of food categories based on person-level consumption. Additionally, ARM explicitly quantifies the strength of associations among food categories, represented as RR in our study. In contrast, dietary pattern analyses do not measure the magnitude of such inter-relationships; rather, they identify latent patterns or clusters of correlated food items. ARM thus provides interpretation similar to food network analysis with weights representing partial correlations rather than RR [57], except that ARM is adapted to large database and is a non-parametric analysis not requiring distributional assumptions of variables.

Grocery retailers and marketing researchers have been using ARM to identify frequently co-purchased products for co-marketing purposes [18, 58]. Such marketing includes the placement of complementary categories adjacent to each other on store shelves [11, 12]. Other marketing tactics, such as simultaneous media advertising and discounting across multiple food categories, may also play a role in influencing purchasing decisions. Identifying co-purchasing patterns using ARM enables further research on these marketing practices and helps identify modifiable drivers of co-purchasing. Insights into co-purchasing associations from ARM can also help identify groups of food items whose sales may shift or fluctuate together in response to economic events or policy interventions (e.g., changes in salty snack sales following a soda tax implementation). Finally, understanding co-purchasing patterns could inform broad-spectrum interventions targeting groups of co-purchased food categories, potentially improving overall dietary patterns more effectively than focusing on single food categories [59, 60].

ARM is a data mining method adapted to large-scale transaction data. Thus, it does not estimate confounder-adjusted associations between food items as in the traditional dietary pattern analysis. Thus, to confirm co-purchasing associations, follow-up regression analysis should be performed as demonstrated in this study to a dataset separate from the one used for ARM to prevent the multiple use of the same data [18, 36]. Such confirmatory regression analysis should account for the correlated nature of longitudinal shopping baskets within shoppers. Following the standard epidemiologic practice, we recommend reporting RR as the main measure of co-purchasing association rather than lift, as the former is easier to interpret the associations [36, 61]. We note that RR estimates can be less than 1.0 for some product pairs that are *substituted* rather than co-purchased, when the two products serve a similar purpose e.g., red meat and meat substitutes. Capturing substitutional association is critical to assess spillover effects of intervention, for example, potential increases in the sales of fruit juice, confectionaries, or water when soda is taxed [13, 59, 62, 63]. However, since substitutional associations have been frequently investigated in the context of price (taxation)-based interventions, we focus on reporting co-purchasing associations in this study. Finally, instead of using ARM, it is possible to apply a series of regression models to estimate all pairwise associations, treating regression modeling as a data mining tool. However, we did not take this approach, as regression models are better suited for etiologic investigations that require careful selection of confounding variables and inspection of model assumptions that should not be automated.

In term of study population, our cardholder population lived in geographic areas (DAs) with a higher proportion of immigrants and people without a high-school diploma, relative to the overall population in the Metropolitan Montréal. Median household income did not notably differ between the two populations, potentially because these stores were utilized by residents with a wide range of area-level income, as the retail chain was not an up-scale nor discount banner and tended to be located on major roads demarcating areas with varying income, with similar spatial accessibility [59, 62]. Most shopping baskets in our data contained a small number of items, with a median of five items. This isconsistent with findings from a previous study on shopping patterns in the U.S., U.K., Canada, New Zealand, Australia, and China, where the median number of items in shopping baskets from sampled supermarkets ranged between four and nine [64]. This pattern may reflect a high prevalence of “fill-in” shopping - quick trips to purchase routinely consumed products such as milk or eggs. Additionally, small basket sizes could be due to shoppers splitting their grocery shopping across multiple chains or stores, resulting in smaller basket sizes per shopping trip. This contrasts with warehouses and supercenters, where baskets tend to be larger in size and shopping trips occur less frequently.

The use of food purchasing data from a loyalty club database has strengths and limitations. Strengths include the automatic collection of longitudinal transactions from a large open cohort of shoppers, often obtained at low cost through a research partnership with a retailer [65]. This is unlike population-representative panel data (e.g., Nielsen Homescan Panel data) generated by household barcode scanners, which are costly to purchase in many countries [66]. The main limitation of cardholder data is non-representativeness, as the data capture shopping patterns from a single retailer. Therefore, the generalizability of our findings is limited to cardholders utilizing a mid-scale supermarket chain in Montréal. However, unlike most studies using cardholder data, our study additionally provided novel insights about the similarities of associations between cardholders and non-cardholders’ baskets, the latter normally unavailable to researchers. Also, most cardholders are not “loyal” to the target chain i.e., utilize multiple supermarket chains (Montréal counts seven chains) and specialty stores such as produce stores, resulting in low frequencies of monthly store visits in our descriptive analysis [67]. Nevertheless, our sensitivity analysis suggests a similar patterning of associations between these loyal and all cardholders and a similar patterning of associations between all cardholders and the subset of cardholders with higher spending. Other limitations of our study include the use of retailer-defined categorization of products, which limits nutritionally relevant classification or profiling of items (e.g., protein, fat, fiber), for instance distinguishing products with and without artificially added sugars e.g., plain vs. sugar-added (flavored) yogurt and cereals, soda vs. diet-soda. Obtaining ingredient compositions or assessing the nutritional quality of food products requires retrospective linking to national food and nutrient databases based on probabilistic and manual matching algorithms [68, 69]. Finally, our data do not contain the exact quantity of each purchased food product (1 vs. 12 bottles of soda). This information will be critical in assessing the etiologic association between purchasing quantities and health outcomes (chronic diseases) for future research.

Future work includes the exploration of co-purchasing patterns involving other food products, in addition to the three food categories examined in this study, including processed and unprocessed red meat. In addition, an ethical framework should be established to guide the use of loyalty card transaction data and other emerging digital data in public health research and surveillance [70]. Specifically, there is a need for criteria to obtain informed consent directly from cardholders when research poses a risk of re-identifying participants. Such research practices requiring rigorous practice to maintain the anonymity of individuals include linking cardholder data at the person level with external datasets and estimating statistics at fine population levels, such as small area estimation [66, 71].

## Conclusions

We explored the co-purchased food categories associated with soda, fresh vegetables, and fresh fruits using transaction data from loyalty card members at a major grocery retailer in Montréal, Canada. Food categories linked to soda included salty snacks and sweet snacks/candies, aligning with unhealthy dietary patterns identified in previous studies. In contrast, food categories co-purchased with fresh vegetables and fresh fruits were those typically found in healthy dietary patterns, such as yogurt and canned vegetables. By using ARM, we were able to quantify the strength of associations among food categories, providing unique insights into the relationships between food categories based on large-scale grocery transaction data.

## Electronic supplementary material

Below is the link to the electronic supplementary material.

Supplementary Material 1

Supplementary Material 2

## Data Availability

The data analyzed in this study were confidential and obtained from a grocery retailer. Requests to access metadata should be directed to the corresponding author. Codes to perform the analysis and prepare data are available publicly: https://github.com/hiroshimamiya/grocery_arm.

## Abbreviations

ARM: Association Rule Mining
CI: Confidence Interval
DA: Dissemination Area
IQR: Interquartile Range
IRB: Institutional Review Board
RR: Risk Ratio or Relative Risk
UPC: Universal Product Code
