# Supplementary material for: Characterizing co-purchased food products with soda, fresh fruits, and fresh vegetables using loyalty card purchasing data in Montréal, Canada, 2015–2017
Source: Int J Behav Nutr Phys Act. 2025 Feb 17;22:19. doi: 10.1186/s12966-024-01701-8 (PMC11834544; doi:10.1186/s12966-024-01701-8)
Supplement: Supplementary file 1 — Supplementary Material 1 [file 12966_2024_1701_MOESM1_ESM.docx]

**Additional File 1**

Contents

[Appendix 1. 3](#_Toc184559861)

[Supplementary Table 1. Summary of basket sizes containing soda, fresh fruits, and fresh vegetables. Grocery loyalty card member data in Montréal, Canada. 2015-2017 4](#_Toc184559862)

[Supplementary Table 2 – Residential socio-demographic and economic characteristics of 15,000 sampled cardholders and all cardholders, compared with the same characteristics in the island of Montreal, 2016 Canadian Census 5](#_Toc184559863)

[Supplementary Figure 1. Top 25 food categories co-purchased with soda, ordered by the value of lift 6](#_Toc184559864)

[Supplementary Figure 2. Top 25 food categories co-purchased with fresh vegetables, ordered by the value of lift 7](#_Toc184559865)

[Supplementary Figure 3. Top 25 food categories co-purchased with fresh fruits, ordered by lift. 8](#_Toc184559866)

[Supplementary Table 3. Main effects and interaction between soda and area-level income and education 9](#_Toc184559867)

[Supplementary Table 4. Main effects and interaction between fresh vegetables and area-level income and education 10](#_Toc184559868)

[Supplementary Table 5. Main effects and interaction between fresh fruits and area-level income and education 11](#_Toc184559869)

[Supplementary Figure 4. Results of association rule mining applied to purchasing data among non-cardholders, showing the risk ratio of top 10 food categories associated with soda. The corresponding risk ratios estimated from cardholder’s baskets in the main analysis are also shown 12](#_Toc184559870)

[Supplementary Figure 5. Results of association rule mining applied to purchasing data among non-cardholders, showing risk ratio of top 10 food categories associated with fresh vegetables. The corresponding risk ratios estimated from cardholder’s baskets in the main analysis are also shown 13](#_Toc184559871)

[Supplementary Figure 6. Results of association rule mining applied to purchasing data among non-cardholders, showing risk ratio of top 10 food categories associated with fresh fruits. The corresponding risk ratios estimated from cardholder’s baskets in the main analysis are also shown 14](#_Toc184559872)

[Supplementary Figure 7. Top 25 food categories co-purchased with soda among a subset of loyal cardholders whose recorded monthly expenditure was greater than 514 Canadian dollars in the target retail chain 15](#_Toc184559873)

[Supplementary Figure 8. Top 25 food categories co-purchased with fresh vegetables among a subset of loyal cardholders whose recorded monthly expenditure was greater than 514 Canadian dollars in the target retail chain 16](#_Toc184559874)

[Supplementary Figure 9. Top 25 food categories co-purchased with fresh fruits among a subset of loyal cardholders whose recorded monthly expenditure was greater than 514 Canadian dollars in the target retail chain 17](#_Toc184559875)

# Appendix 1.

Minimum support is the threshold frequency (support) of the combination of food categories among baskets, which is used to exclude infrequently occurring co-purchasing combinations from the computational process of association mining. The other parameter, minimum confidence, is the threshold for the strength of co-purchasing associations, below which ARM does not estimate and rank co-purchasing associations. Confidence represents the conditional probability of item X appearing in a basket given the presence of another item Y, thus P(X|Y). Thus, setting a lower value for minimum confidence results in ARM estimating weak co-purchasing associations, with the cost of an increased computational time. We set minimum support to 1% i.e., excluding category combinations whose prevalence among baskets is below 1%. Such low values comprehensively search associations, even though relatively infrequent associations with less public health importance would be reported. Minimum confidence was set to 5%, indicating that ARM will select category pairs with P(X|Y) greater than 0.05, where Y indicates one of our target food categories: soda, fresh vegetables, and fresh fruits. The low value of confidence allowed searching co-purchasing pairs that are potentially important for public health with a slightly increased computational time.

# **Supplementary Table 1.** Summary of basket sizes containing soda, fresh fruits, and fresh vegetables. Grocery loyalty card member data in Montréal, Canada. 2015-2017

|  | Soda | |  | Fresh fruits | |  | Fresh vegetables | | | |
| --- | --- | --- | --- | --- | --- | --- | --- | --- | --- | --- |
| Basket summary | Median | IQR |  | Median | IQR |  | | Median | IQR | |
| Number of categories ^a^ | 8 | (4, 14) |  | 7 | (4, 12) |  | | 8 | | (5, 12) |
| product  quantities ^b^ | 12 | (6, 24) |  | 16 | (7, 40) |  | | 19 | | (8, 52) |
| Dollar  spending | 43.5 | (20.3, 88.0) |  | 41.8 | (21.9, 76.8) |  | | 43.1 | | (23.1, 78.2) |

Abbreviation: IQR, interquartile range

^a^ Number of distinct categories within baskets

^b^ Number of purchased food products within baskets

Basket represents a list of items purchased in a single transaction (shopping trip)

# Supplementary Table 2 – Residential socio-demographic and economic characteristics of 15,000 sampled cardholders and all cardholders, compared with the same characteristics in the island of Montreal, 2016 Canadian Census

|  | Sample of cardholders | |  | All cardholders | |  | Census population | |
| --- | --- | --- | --- | --- | --- | --- | --- | --- |
| Population Characteristics | Median | IQR |  | Median | IQR |  | Median | IQR |
| Education ^a^ | 13.5 | 8.2 - 20.4 |  | 13.5 | 8.1 - 20.7 |  | 16.2 | 9.8 - 24.0 |
| Median family income (in 10,000 Canadian dollars) | 51.5 | 42.5 - 67.0 |  | 51.5 | 42.4 - 65.8 |  | 52.4 | 43.3 - 67.4 |
| % employment | 59.7 | 52.9 - 66.7 |  | 59.7 | 52.8-66.7 |  | 58.5 | 52.0-65.3 |
| % immigrant | 26.6 | 18.3 - 38.4 |  | 26.8 | 18.3 - 38.7 |  | 31.4 | 20.6 - 44.9 |
| Mean age | 39.8 | 37.0 - 43.0 |  | 39.8 | 37.0 - 43.0 |  | 39.7 | 37.1 - 42.6 |
| Mean family size | 2.8 | 2.6-3.0 |  | 2.8 | 2.6-3.0 |  | 2.9 | 2.7-3.1 |

Abbreviation: IQR, interquartile range

Variables represent aggregated census characteristics of census dissemination areas in which cardholders lived, thus being ecological (area-level) characteristics

^a^ % of residents without high-school diploma or certificate among age greater than 25

^b^ % of residents with part- or full-time work among those classified as labor force


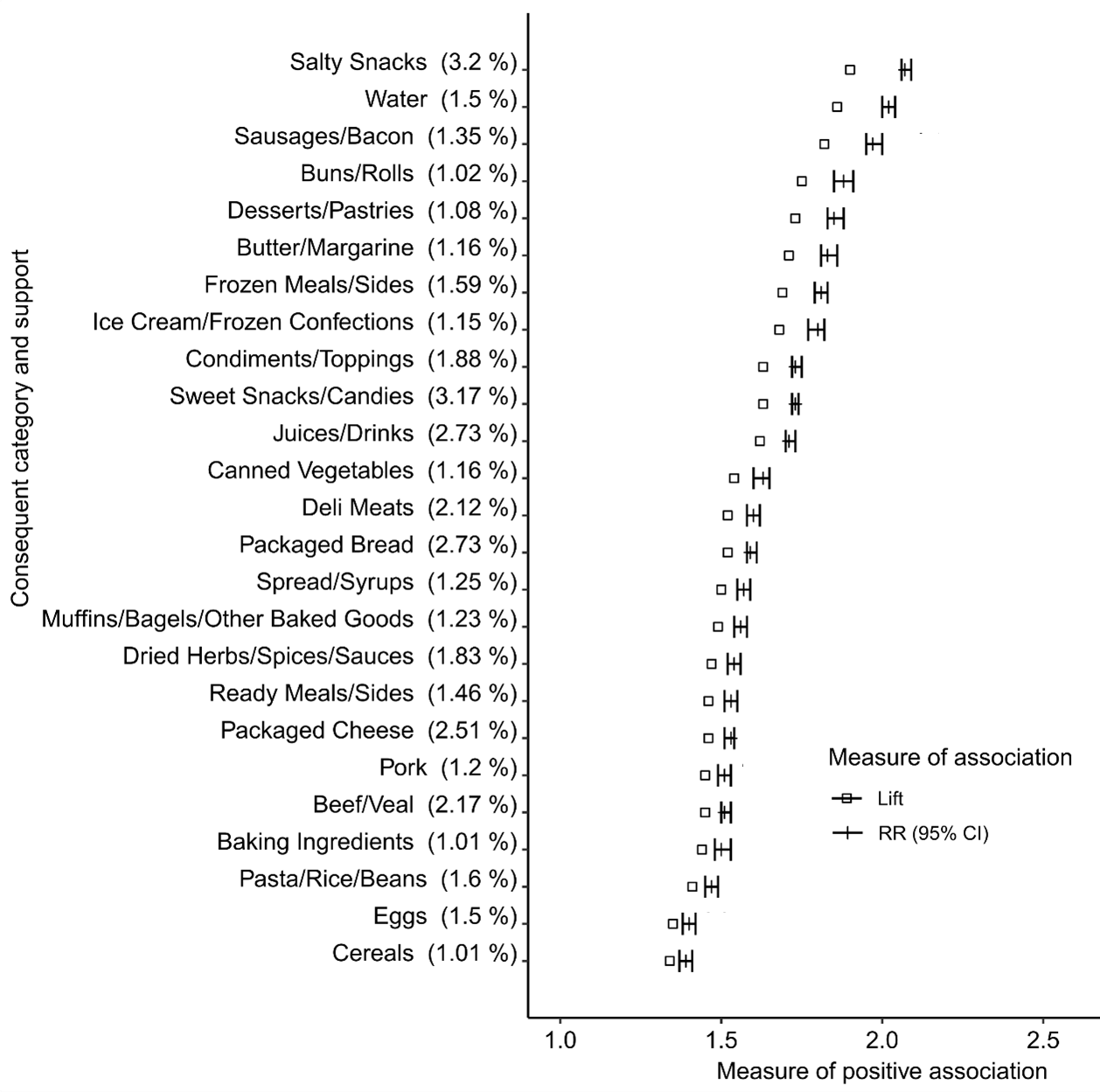


# Supplementary Figure 1. Top 25 food categories co-purchased with soda, ordered by the value of lift

Abbreviations: RR; Risk Ratio, CI; Confidence Interval

Support indicates the percent joint frequency of the antecedent (soda) and each of the consequents


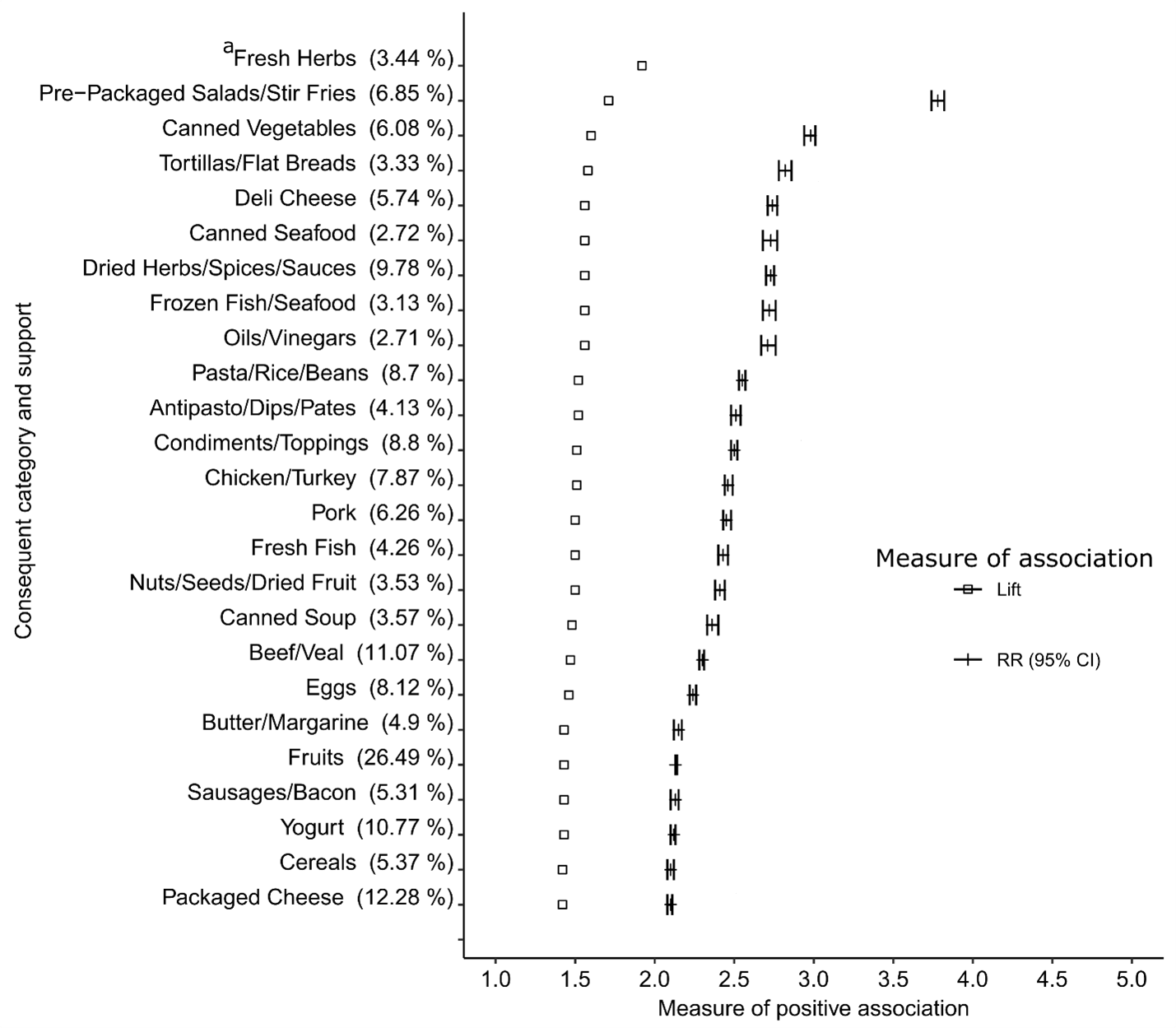


# Supplementary Figure 2. Top 25 food categories co-purchased with fresh vegetables, ordered by the value of lift

^a^ RR and its 95%CI of the Fresh Herbs category is not displayed, as the estimates (RR=6.56, 95%CI;6.43-6.70) is outside the scale of the x-axis

Support indicates the percent joint frequency of the antecedent (fresh vegetable) and each of the consequents


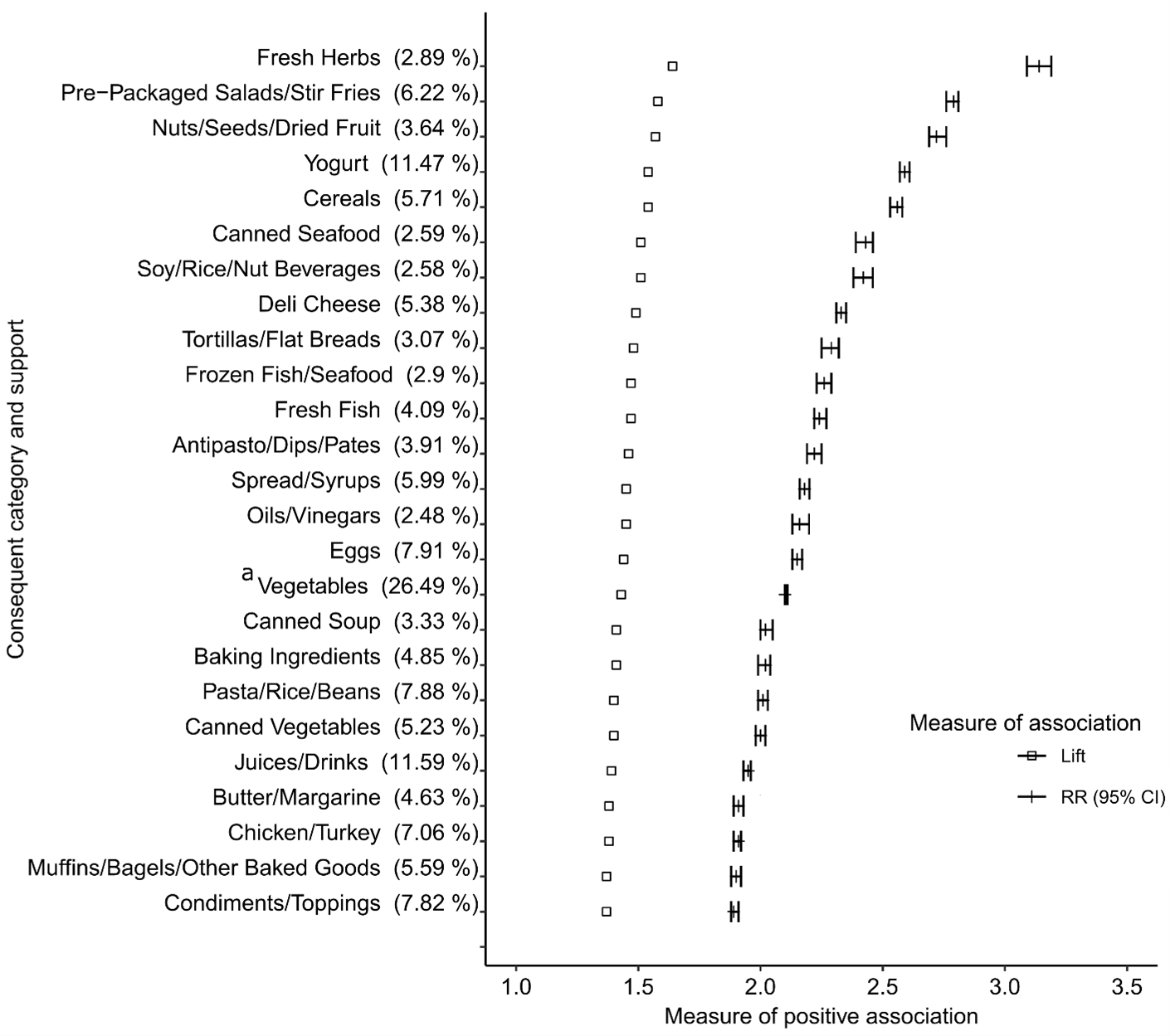


# Supplementary Figure 3. Top 25 food categories co-purchased with fresh fruits, ordered by lift.

Abbreviations: RR; Risk Ratio, CI; Confidence Interval

Support indicates the joint frequency of antecedent (fresh fruits) and each of the consequents, in percent

# Supplementary Table 3. Main effects and interaction between soda and area-level income and education

| **Outcome** | **Interaction or main effect ^a^** | **OR**  **(95% CI)^b^** | **P-value** |
| --- | --- | --- | --- |
|  |  |  |  |
| Salty Snacks | Soda **X** Income | 0.99 (0.97, 1.02) | 0.608671 |
|  | Soda **X** Education | 1.00 (0.98, 1.03) | 0.755664 |
|  | Soda* | 2.00 (1.98, 2.05) | <1.00E-10 |
|  | Income* | 1.07 (1.04, 1.10) | 4.93E-08 |
|  | Education | 0.99 (0.96,1.02) | 0.583591 |
| Sweet snacks and candies | Soda **X** Income | 1.01 (0.99, 1.03) | 0.289531 |
|  | Soda **X** Education* | 1.04 (1.03, 1.06) | 0.000022 |
|  | Soda* | 1.34 (1.32, 1.36) | <1.00E-10 |
|  | Income | 1.02 (0.99, 1.05) | 0.174374 |
|  | Education* | 1.04 (1.00, 1.06) | 0.012549 |
| Juices and drinks | Soda **X** Income* | 1.05 (1.04, 1.08) | 0.000001 |
|  | Soda **X** Education* | 1.03 (1.02, 1.05) | 0.000657 |
|  | Soda* | 1.47 (1.44, 1.50) | <1.00E-10 |
|  | Income | 0.98 (0.95, 1.01) | 0.195162 |
|  | Education* | 0.91 (0.88, 0.93) | <1.00E-10 |

Abbreviations: OR, Odds Ratio; 95% CI, 95% Confidence Interval

^a^ “X” indicates interaction

* Statistically conclusive result (p-value is less than the critical value of 0.05, and 95% confidence interval not crossing the null value of OR=1.0)

Income and education variables are aggregated census measures at the level of the census dissemination area in which cardholders resided. These measures represent median family income and the proportion of residents without a high-school diploma, respectively. Both continuous variables are standardized: mean centered and scaled by 1-unit standard deviation. The variables “Soda” is the binary indicator of soda purchasing.

# Supplementary Table 4. Main effects and interaction between fresh vegetables and area-level income and education

| **Outcome** | **Interaction or main effect^a^** | **OR**  **(95% CI)^b^** | **P-value** |
| --- | --- | --- | --- |
|  |  |  |  |
| Pre-Packaged Salads/Stir Fries | Fresh vegetables **X** Income * | 1.02 (1.01, 1.04) | 0.007912 |
|  | Fresh vegetables **X** Education * | 0.96 (0.94, 0.98) | 0.000010 |
|  | Fresh vegetables * | 2.63 (2.59 2.68) | <1.00E-10 |
|  | Income * | 1.15 (1.12 1.20) | <1.00E-10 |
|  | Education * | 0.89 (0.86 0.92) | <1.00E-10 |
| Canned Vegetables | Fresh vegetables **X** Income | 0.99 (0.97, 1.01) | 0.331972 |
|  | Fresh vegetables **X** Education* | 0.98 (0.96, 1.00) | 0.019090 |
|  | Fresh vegetables* | 1.86 (1.83 1.90) | <1.00E-10 |
|  | Income * | 0.97 (0.94 1.00) | 0.028298 |
|  | Education | 1.02 (0.99 1.05) | 0.164189 |
| Deli Cheese | Fresh vegetables **X** Income | 1.02 (1.00, 1.03) | 0.083309 |
|  | Fresh vegetables **X** Education* | 0.97 (0.95, 0.99) | 0.007549 |
|  | Fresh vegetables* | 1.61 (1.59 1.64) | <1.00E-10 |
|  | Income * | 1.10 (1.07 1.14) | <1.00E-10 |
|  | Education* | 0.87 (0.83 0.90) | 3.98E-08 |

Abbreviations: OR, Odds Ratio; 95% CI, 95% Confidence Interval

^a^ “X” indicates interaction

* Statistically conclusive result (p-value is less than the critical value of 0.05, and 95% confidence interval not crossing the null value of OR=1.0)

Income and education variables are aggregated census measures at the level of the census dissemination area in which cardholders resided. These measures represent median family income and the proportion of residents without a high-school diploma, respectively. Both continuous variables are standardized: mean centered and scaled by 1-unit standard deviation. The variables “Fresh vegetables” is the binary indicator of fresh vegetable purchasing.

# Supplementary Table 5. Main effects and interaction between fresh fruits and area-level income and education

| **Outcome** | **Interaction or main effect ^a^** | **OR**  **(95% CI) ^b^** | **P-value** |
| --- | --- | --- | --- |
|  |  |  |  |
| Pre-Packaged Salads/Stir Fries | Fresh fruits **X** Income* | 1.02 (1.00, 1.04) | 0.019330 |
|  | Fresh fruits **X** Education* | 0.97 (0.95, 0.99) | 0.000264 |
|  | Fresh fruits* | 1.89 (1.86 1.93) | <1.00E-10 |
|  | Income* | 1.17 (1.13 1.21) | <1.00E-10 |
|  | Education* | 0.88 (0.85 0.92) | <1.00E-10 |
| Nuts/Seeds/Dried Fruit | Fresh fruits **X** Income* | 1.02 (1.01, 1.04) | 0.001851 |
|  | Fresh fruits **X** Education* | 1.03 (1.02, 1.05) | 0.000033 |
|  | Fresh fruits | 1.93 (1.91 1.96) | <1.00E-10 |
|  | Income | 1.02 (1.00 1.06) | 0.133996 |
|  | Education* | 0.81 (0.78 0.84) | <1.00E-10 |
| Yogurt | Fresh fruits **X** Income* | 1.02 (1.00, 1.04) | 0.014930 |
|  | Fresh fruits **X** Education | 1.02 (1.00, 1.04) | 0.064431 |
|  | Fresh fruits* | 1.62 (1.60 1.65) | <1.00E-10 |
|  | Income | 1.00 (0.96 1.04) | 0.965686 |
|  | Education* | 0.87 (0.83 0.90) | <1.00E-10 |

Abbreviations: OR, Odds Ratio; 95% CI, 95% Confidence Interval

^a^ “X” indicates interaction

* Statistically conclusive result (p-value is less than the critical value of 0.05, and 95% confidence interval not crossing the null value of OR=1.0)

Income and education variables are aggregated census measures at the level of the census dissemination area in which cardholders resided. These measures represent median family income and the proportion of residents without a high-school diploma, respectively. Both continuous variables are standardized: mean centered and scaled by 1-unit standard deviation. The variables “Fresh fruits” is the binary indicator of fresh fruits purchasing.


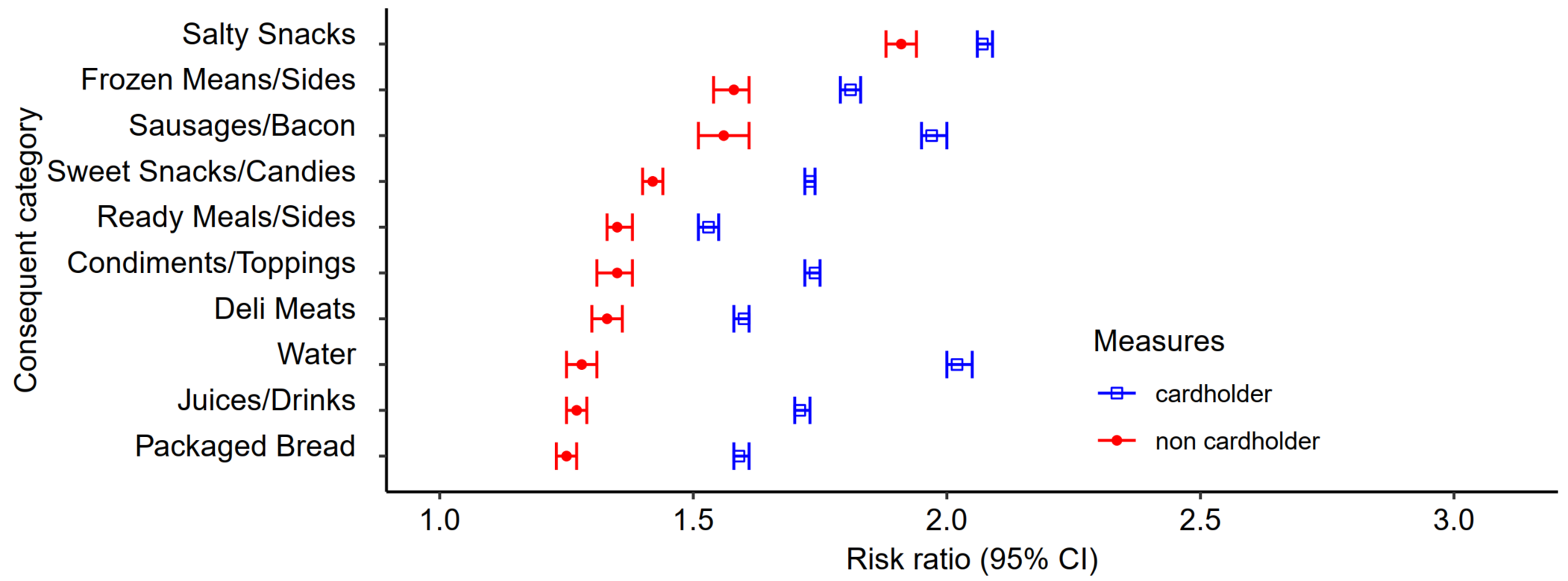


# Supplementary Figure 4. Results of association rule mining applied to purchasing data among non-cardholders, showing the risk ratio of top 10 food categories associated with soda. The corresponding risk ratios estimated from cardholder’s baskets in the main analysis are also shown


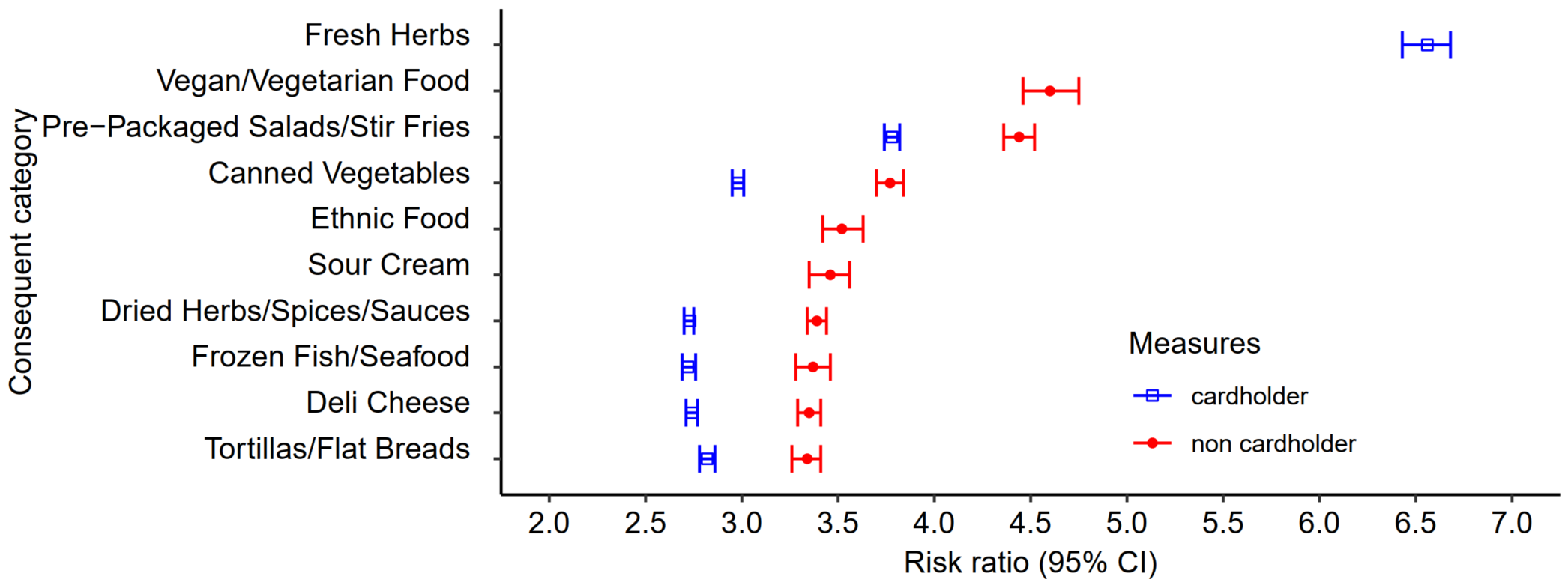


# Supplementary Figure 5. Results of association rule mining applied to purchasing data among non-cardholders, showing risk ratio of top 10 food categories associated with fresh vegetables. The corresponding risk ratios estimated from cardholder’s baskets in the main analysis are also shown

Missing estimates from cardholders for vegan/vegetarian food, ethnic food, and sour cream indicate that these categories are not raked to the top 25 categories of cardholders in the main analysis


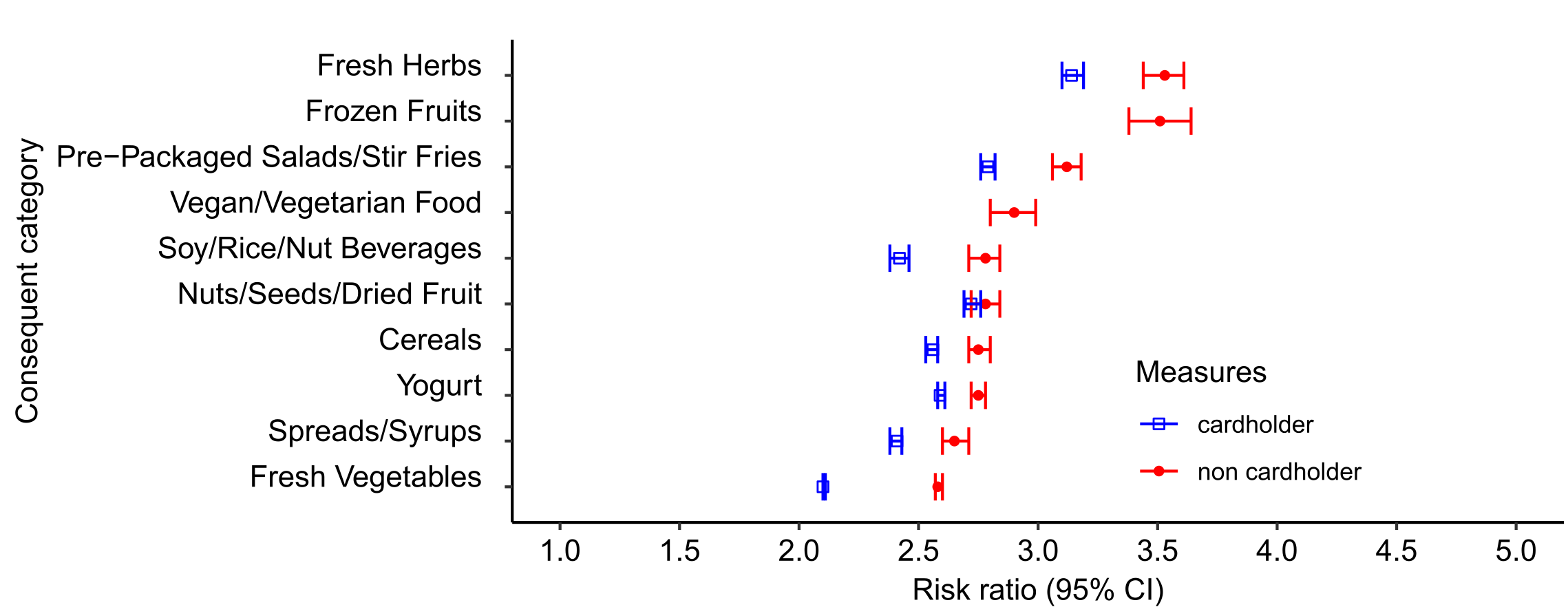


# Supplementary Figure 6. Results of association rule mining applied to purchasing data among non-cardholders, showing risk ratio of top 10 food categories associated with fresh fruits. The corresponding risk ratios estimated from cardholder’s baskets in the main analysis are also shown

Missing estimates from cardholders for frozen fruits and began/vegetarian food indicate that these categories are not raked to the top 25 categories of cardholders


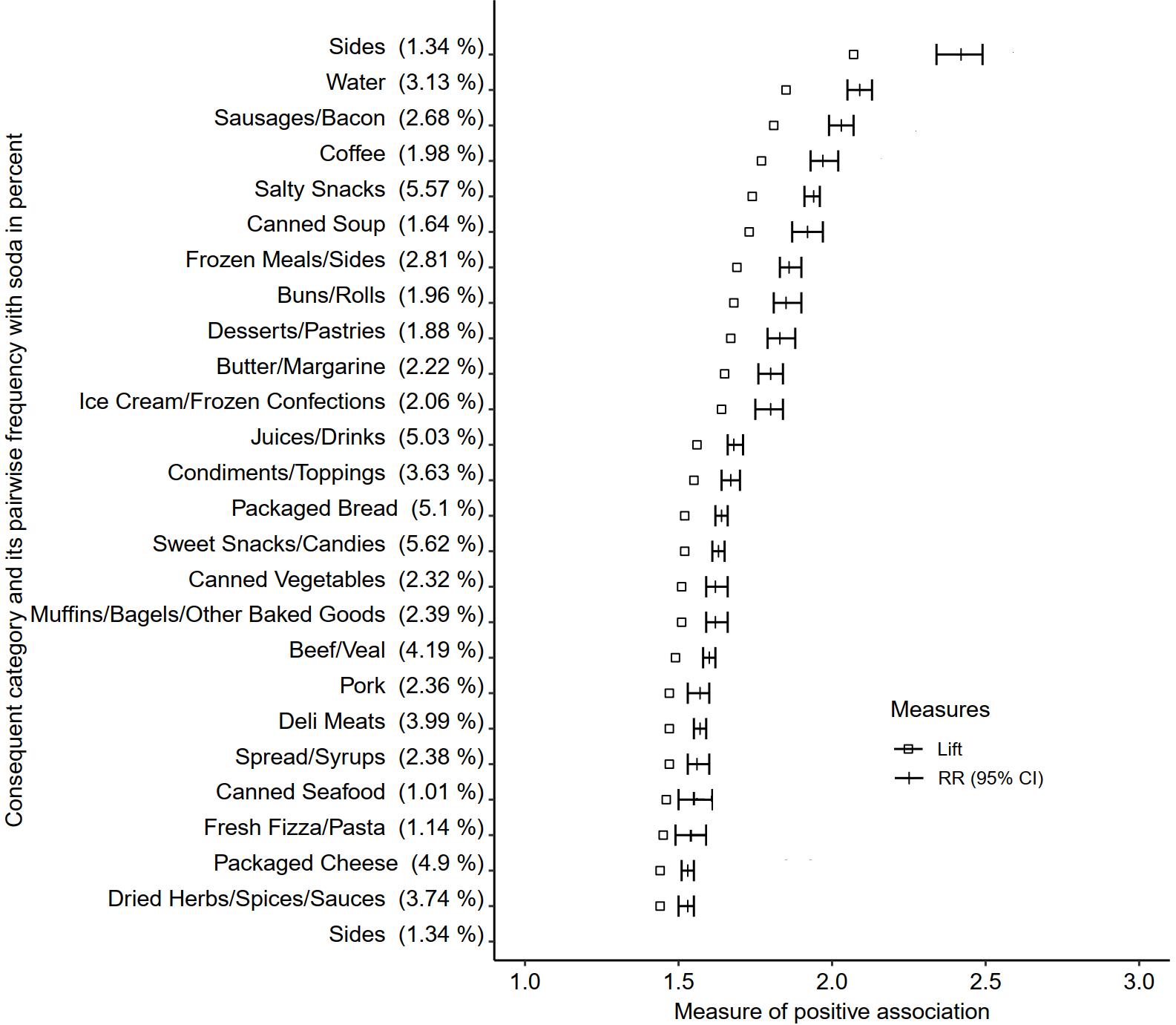


Supplementary Figure 7. Top 25 food categories co-purchased with soda among a subset of loyal cardholders whose recorded monthly expenditure was greater than 514 Canadian dollars in the target retail chain

Abbreviations: RR; Relative Risk, CI; Confidence Interval


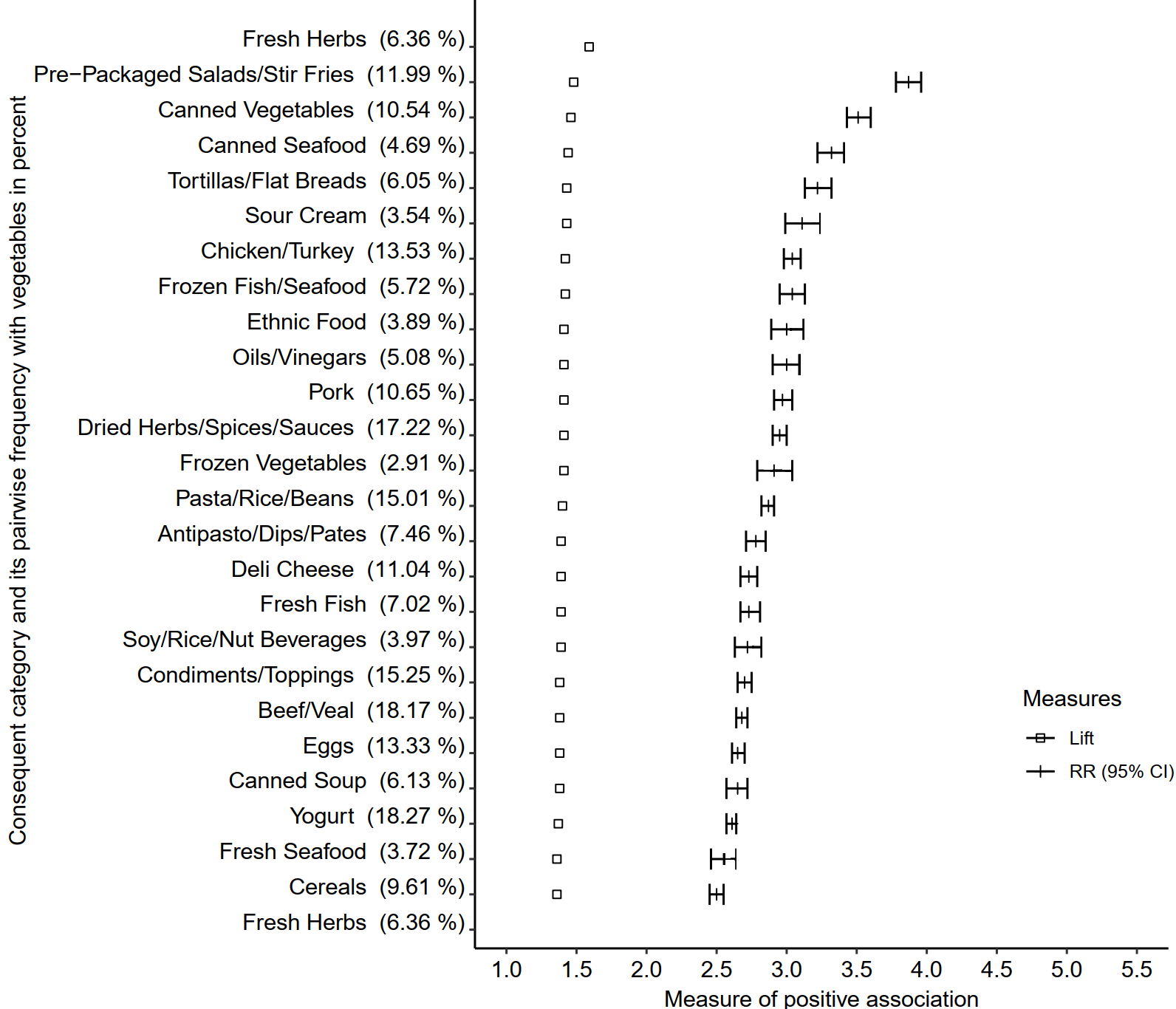


Supplementary Figure 8. Top 25 food categories co-purchased with fresh vegetables among a subset of loyal cardholders whose recorded monthly expenditure was greater than 514 Canadian dollars in the target retail chain

Abbreviations: RR; Relative Risk, CI; Confidence Interval

RR and their 95%CI of the Fresh Herbs category are not displayed, as the estimate (RR=6.49, 95%CI;6.24-6.76) are outside the scale of the x-axis


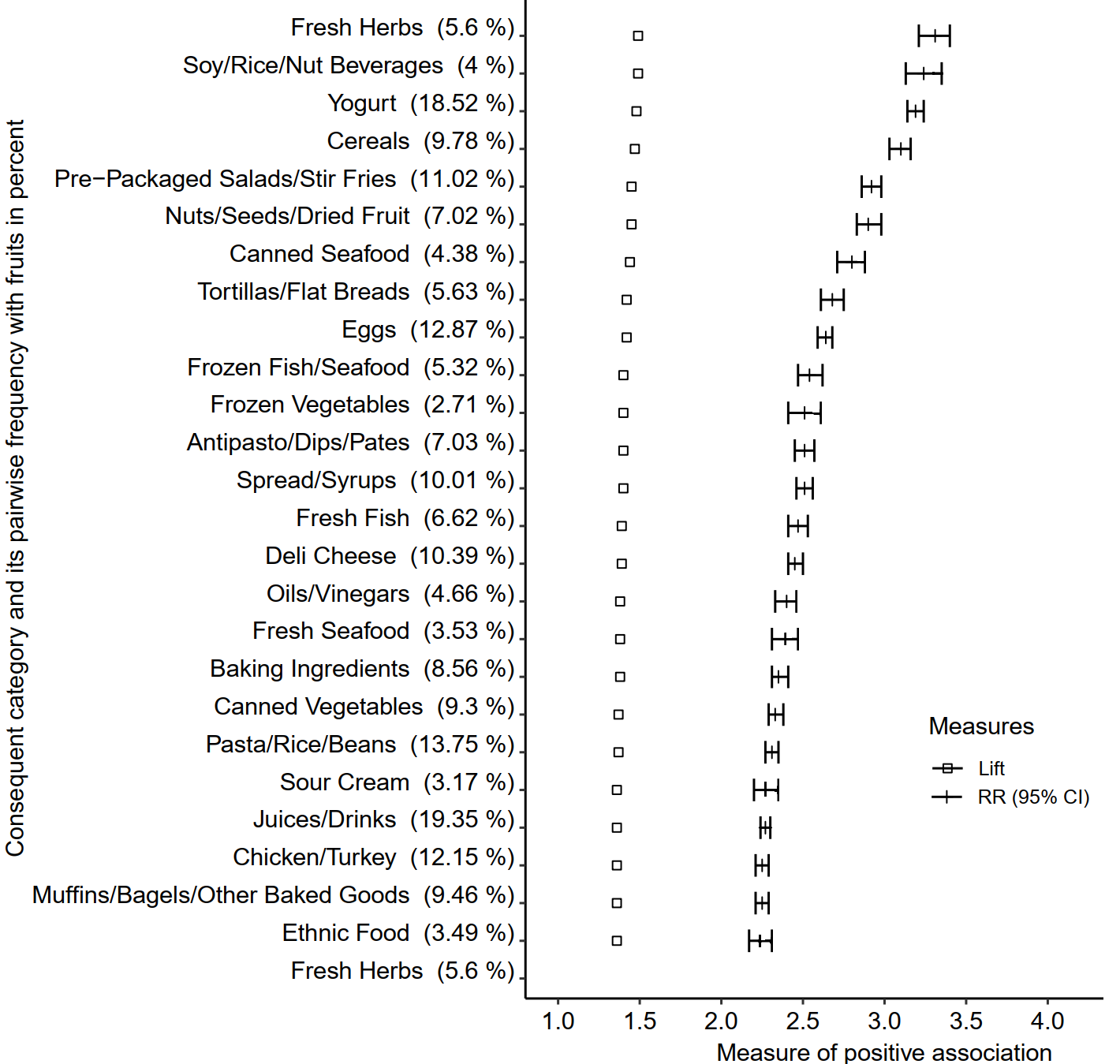


Supplementary Figure 9. Top 25 food categories co-purchased with fresh fruits among a subset of loyal cardholders whose recorded monthly expenditure was greater than 514 Canadian dollars in the target retail chain

Abbreviations: RR; Relative Risk, CI; Confidence Interval
