# Supplementary material for: Characterizing co-purchased food products with soda, fresh fruits, and fresh vegetables using loyalty card purchasing data in Montréal, Canada, 2015–2017
Source: Int J Behav Nutr Phys Act. 2025 Feb 17;22:19. doi: 10.1186/s12966-024-01701-8 (PMC11834544; doi:10.1186/s12966-024-01701-8)
Supplement: Supplementary file 2 — Supplementary Material 2 [file 12966_2024_1701_MOESM2_ESM.pdf]

**Additional File 2.**

**Table 1. STROBE-nut: An extension of the STROBE statement for nutritional epidemiology**

Lachat C et al. (2016) STrengthening the Reporting of OBservational studies in Epidemiology – Nutritional Epidemiology (STROBE-nut): an extension of the STROBE statement. Plos Medicine 13(6) http://dx.doi.org/10.1371/journal.pmed.1002036 pdf or online version.

| Item | Item nr | STROBE recommendations | Extension for Nutritional Epidemiology studies (STROBE-nut) | Reported on page # |
|---|---|---|---|---|
| **Title and abstract** | 1 | (a) Indicate the study's design with a commonly used term in the title or the abstract.<br>(b) Provide in the abstract an informative and balanced summary of what was done and what was found. | **nut-1** State the dietary/nutritional assessment method(s) used in the title, abstract, or keywords. | (a), (b), and Nut-1. Title Page (Page 1) and Page 2 (Abstract) |
| **Introduction** | | | | |
| Background rationale | 2 | Explain the scientific background and rationale for the investigation being reported. | | Pages 4 and 5 |

| Item | Item nr | STROBE recommendations | Extension for Nutritional Epidemiology studies (STROBE-nut) | Reported on page # |
|---|---|---|---|---|
| Objectives | 3 | State specific objectives, including any pre-specified hypotheses. | | Objectives 1 and 2: Pages 5 and 6. |
| **Methods** | | | | |
| Study design | 4 | Present key elements of study design early in the paper. | | Page 6. |
| Settings | 5 | Describe the setting, locations, and relevant dates, including periods of recruitment, exposure, follow-up, and data collection. | **nut-5** Describe any characteristics of the study settings that might affect the dietary intake or nutritional status of the participants, if applicable. | Pages 6 and 7. |
| Participants | 6 | a) Cohort study—Give the eligibility criteria, and the sources and methods of selection of | **nut-6** Report particular dietary, physiological or nutritional characteristics that were considered | (a), (b) and Nut-6. Pages 6- 7 and Figure 1 for the description of loyalty card cohort, exclusion criteria, and the size of the retail chain. |

| Item | Item nr | STROBE recommendations | Extension for Nutritional Epidemiology studies (STROBE-nut) | Reported on page # |
|---|---|---|---|---|
| | | participants. Describe methods of follow-up.<br><br>Case-control study—Give the eligibility criteria, and the sources and methods of case ascertainment and control selection. Give the rationale for the choice of cases and controls.<br><br>Cross-sectional study—Give the eligibility criteria, and the sources and methods of selection of participants.<br><br>(b) Cohort study—For matched studies, give matching criteria and number of exposed and unexposed. | when selecting the target population. | |

| Item | Item nr | STROBE recommendations | Extension for Nutritional Epidemiology studies (STROBE-nut) | Reported on page # |
|---|---|---|---|---|
| | | Case-control study—For matched studies, give matching criteria and the number of controls per case. | | |
| Variables | 7 | Clearly define all outcomes, exposures, predictors, potential confounders, and effect modifiers. Give diagnostic criteria, if applicable. | **nut-7.1** Clearly define foods, food groups, nutrients, or other food components.<br><br>**nut-7.2** When using dietary patterns or indices, describe the methods to obtain them and their nutritional properties. | Nut-7.1. Page 8 and Table 1 (food groups)<br><br>Nut-7.2. Page 9 (analysis of purchasing patterns).<br><br>Page 11-12 for longitudinal regression (Exposure, outcome, confounders and effect measure modifiers) |
| Data sources - measurements | 8 | For each variable of interest, give sources of data and details of methods of assessment (measurement). | **nut-8.1** Describe the dietary assessment method(s), e.g., portion size | Page 7 (description of scanner data).<br><br>Nut 8.2, 8.3, 84, 8.6 N/A. Due to the nature of retailer-defined food category in this study, food composition data |

| Item | Item nr | STROBE recommendations | Extension for Nutritional Epidemiology studies (STROBE-nut) | Reported on page # |
|---|---|---|---|---|
| | | Describe comparability of assessment methods if there is more than one group. | estimation, number of days and items recorded, how it was developed and administered, and how quality was assured. Report if and how supplement intake was assessed.<br><br>**nut-8.2** Describe and justify food composition data used. Explain the procedure to match food composition with consumption data. Describe the use of conversion factors, if applicable.<br><br>**nut-8.3** Describe the nutrient | was not used. Dietary reference nor nutritional biomarkers were not used in this study.<br><br>Nut 8.1. N/A Portion size was not available in this scanner data. The quantity was recorded as the number of items purchased (beginning of Page 9).<br><br>Nut 8.5. Page 12 - The timing of the assessment of dietary intake is not relevant in this study that addressed purchasing. However, the influencing factors of purchasing were described in the description of regression variables (confounders, Page 12). |

| Item | Item nr | STROBE recommendations | Extension for Nutritional Epidemiology studies (STROBE-nut) | Reported on page # |
|---|---|---|---|---|
| | | | requirements, recommendations, or dietary guidelines and the evaluation approach used to compare intake with the dietary reference values, if applicable.<br><br>**nut-8.4** When using nutritional biomarkers, additionally use the STROBE Extension for Molecular Epidemiology (STROBE-ME). Report the type of biomarkers used and their usefulness as dietary exposure markers. | |

| Item | Item nr | STROBE recommendations | Extension for Nutritional Epidemiology studies (STROBE-nut) | Reported on page # |
|---|---|---|---|---|
| | | | **nut-8.5** Describe the assessment of nondietary data (e.g., nutritional status and influencing factors) and timing of the assessment of these variables in relation to dietary assessment.<br><br>**nut-8.6** Report on the validity of the dietary or nutritional assessment methods and any internal or external validation used in the study, if applicable. | |

| Item | Item nr | STROBE recommendations | Extension for Nutritional Epidemiology studies (STROBE-nut) | Reported on page # |
| --- | --- | --- | --- | --- |
| Bias | 9 | Describe any efforts to address potential sources of bias. | **nut-9** Report how bias in dietary or nutritional assessment was addressed, e.g., misreporting, changes in habits as a result of being measured, or data imputation from other sources | Page 12-13 (sensitivity analysis for frequent and non-frequent users and non-cardholders). |
| Study Size | 10 | Explain how the study size was arrived at. | | Pages 7 and 8 (Exclusion criteria and sample splitting for Objectives 1 and 2) |
| Quantitative variables | 11 | Explain how quantitative variables were handled in the analyses. If applicable, describe which groupings were chosen and why. | **nut-11** Explain the categorization of dietary/nutritional data (e.g., use of N-tiles and handling of non-consumers) and the choice of reference category, if applicable. | Page 8 (Objective 1 – association rule mining) and Page 11 (Objective 2 – regression analysis) |

| Item | Item nr | STROBE recommendations | Extension for Nutritional Epidemiology studies (STROBE-nut) | Reported on page # |
|---|---|---|---|---|
| Statistical<br><br>Methods | 12 | (a) Describe all statistical methods, including those used to control for confounding<br><br>(b) Describe any methods used to examine subgroups and interactions.<br><br>(c) Explain how missing data were addressed.<br><br>(d) Cohort study—If applicable, explain how loss to follow-up was addressed.<br><br>Case-control study—If applicable, explain how matching of cases and controls was addressed.<br><br>Cross-sectional study—If applicable, describe analytical methods | **nut-12.1** Describe any statistical method used to combine dietary or nutritional data, if applicable.<br><br>**nut-12.2** Describe and justify the method for energy adjustments, intake modeling, and use of weighting factors, if applicable.<br><br>**nut-12.3** Report any adjustments for measurement error, i.e,. from a validity or calibration study. | (a) Page 9 – 11.<br><br>(b) Page 12<br><br>(c) N/A<br><br>(d) N/A. Exclusion time is not relevant to this open cohort that does not have a well-defined withdrawal time and definition (Page 7).<br><br>(e ) Page 12-13<br><br>Nut 12.1 N/A<br><br>Nut 12.2 N/A<br><br>Nut 12.3 N/A |

| Item | Item nr | STROBE recommendations | Extension for Nutritional Epidemiology studies (STROBE-nut) | Reported on page # |
|---|---|---|---|---|
| | | taking account of sampling strategy.<br><br>(e) Describe any sensitivity analyses. | | |
| **Results** | | | | |
| Participants | 13 | (a) Report the numbers of individuals at each stage of the study—e.g., numbers potentially eligible, examined for eligibility, confirmed eligible, included in the study, completing follow-up, and analyzed.<br><br>(b) Give reasons for non-participation at each stage.<br><br>(c) Consider use of a flow diagram. | **nut-13** Report the number of individuals excluded based on missing, incomplete or implausible dietary/nutritional data. | (a) Page 13 and Figure 1.<br><br>(b) Page (8, exclusion criteria) and Figure 1.<br><br>(c) Figure 1. |
| Descriptive data | 14 | (a) Give characteristics of study participants | **nut-14** Give the distribution of | (a) Supplementary Table 2 and Page 14. |

| Item | Item nr | STROBE recommendations | Extension for Nutritional Epidemiology studies (STROBE-nut) | Reported on page # |
|---|---|---|---|---|
| | | (e.g., demographic, clinical, social) and information on exposures and potential confounders<br><br>(b) Indicate the number of participants with missing data for each variable of interest<br><br>(c) Cohort study—Summarize follow-up time (e.g., average and total amount) | participant characteristics across the exposure variables if applicable. Specify if food consumption of total population or consumers only were used to obtain results. | (b) N/A. Transaction data and census areal variables did not contain missing data, except a very small proportion of negative transactions that might indicate returned food items (Page 8).<br><br>(c) N/A. This open cohort does not have well-defined follow-up time as explained in Page 7. |
| Outcome data | 15 | Cohort study—Report numbers of outcome events or summary measures over time.<br><br>Case-control study—Report numbers in each exposure category, or | | Figure 2 and Page 13. |

| Item | Item nr | STROBE recommendations | Extension for Nutritional Epidemiology studies (STROBE-nut) | Reported on page # |
| --- | --- | --- | --- | --- |
| | | summary measures of exposure.<br><br>Cross-sectional study—Report numbers of outcome events or summary measures. | | |
| Main results | 16 | (a) Give unadjusted estimates and, if applicable, confounder-adjusted estimates and their precision (e.g., 95% confidence interval).<br><br>Make clear which confounders were adjusted for and why they were included.<br><br>(b) Report category boundaries when continuous variables were categorized. | **nut-16** Specify if nutrient intakes are reported with or without inclusion of dietary supplement intake, if applicable. | (a)Pages 14 and 15 (unadjusted associations in Objective 1) and Page 15-16 (adjusted estimates in Objective 2), with corresponding figures.<br><br>(b) N/A<br><br>(c) In this study, we converted odds ratio into risk ratio.<br><br>Nut16. N/A |

| Item | Item nr | STROBE recommendations | Extension for Nutritional Epidemiology studies (STROBE-nut) | Reported on page # |
|---|---|---|---|---|
| | | (c) If relevant, consider translating estimates of relative risk into absolute risk for a meaningful time period. | | |
| Other analyses | 17 | Report other analyses done—e.g., analyses of subgroups and interactions and sensitivity analyses. | **nut-17** Report any sensitivity analysis (e.g., exclusion of misreporters or outliers) and data imputation, if applicable. | Pages 15 and 16 (sensitivity analyses) |
| **Discussion** | | | | |
| Key results | 18 | Summarize key results with reference to study objectives. | | Page 17 |
| Limitation | 19 | Discuss limitations of the study, taking into account sources of potential bias or imprecision. Discuss | **nut-19** Describe the main limitations of the data sources and assessment | Page 22 |

| Item | Item nr | STROBE recommendations | Extension for Nutritional Epidemiology studies (STROBE-nut) | Reported on page # |
|---|---|---|---|---|
| | | both direction and magnitude of any potential bias. | methods used and implications for the interpretation of the findings. | |
| Interpretation | 20 | Give a cautious overall interpretation of results considering objectives, limitations, multiplicity of analyses, results from similar studies, and other relevant evidence. | **nut-20** Report the nutritional relevance of the findings, given the complexity of diet or nutrition as an exposure. | Page 17-19 |
| Generalizability | 21 | Discuss the generalizability (external validity) of the study results. | | Page 21-22 |
| **Other information** | | | | |
| Funding | 22 | Give the source of funding and the role of the funders for the present study and, if applicable, for the original study on which | | Page 23 (Declarations) |

| Item | Item nr | STROBE recommendations | Extension for Nutritional Epidemiology studies (STROBE-nut) | Reported on page # |
|---|---|---|---|---|
| | | the present article is based. | | |
| *Ethics* | | | **nut-22.1** Describe the procedure for consent and study approval from ethics committee(s). | Page 23 (Declarations) |
| *Supplementary material* | | | **nut-22.2** Provide data collection tools and data as online material or explain how they can be accessed. | Separate supplementary file contains supplementary appendix, files, and tables. |
